# The dual action of glioma-derived exosomes on neuronal activity: synchronization and disruption of synchrony

**DOI:** 10.1038/s41419-022-05144-6

**Published:** 2022-08-13

**Authors:** Renza Spelat, Nie Jihua, Cesar Adolfo Sánchez Triviño, Simone Pifferi, Diletta Pozzi, Matteo Manzati, Simone Mortal, Irene Schiavo, Federica Spada, Melania Eva Zanchetta, Tamara Ius, Ivana Manini, Irene Giulia Rolle, Pietro Parisse, Ana P. Millán, Ginestra Bianconi, Fabrizia Cesca, Michele Giugliano, Anna Menini, Daniela Cesselli, Miran Skrap, Vincent Torre

**Affiliations:** 1 International School for Advanced Studies (SISSA), via Bonomea 265, Trieste, 34136 Italy; 2 Institute of Materials (IOM-CNR), Area Science Park, Basovizza, 34149 Trieste, Italy; 3 Neurosurgery Unit, Department of Neurosciences, Santa Maria della Misericordia University Hospital, 33100 Udine, Italy; 4 Università degli studi di Udine, Istituto di Anatomia Patologica, ASUIUD, Udine, Italy; 5 Department of Clinical Neurophysiology and MEG Center, Amsterdam Neuroscience, Amsterdam UMC, Vrije Universiteit Amsterdam, De Boelelaan 1117, Amsterdam, The Netherlands; 6 School of Mathematical Sciences, Queen Mary University of London, Mile End Road, E1 4NS London, UK; 7 Alan Turing Institute, The British Library, 96 Euston Road, London, UK; 8 Department of Life Sciences, University of Trieste, 34127 Trieste, Italy; 9 SOC Neurochirurgia, Az. Ospedaliera Sanitaria Integrata, Udine, Italy; 10 Biovalley Systems & Solutions S.r.l., 34148 Trieste, Italy

**Keywords:** Neuroscience, Preclinical research

## Abstract

Seizures represent a frequent symptom in gliomas and significantly impact patient morbidity and quality of life. Although the pathogenesis of tumor-related seizures is not fully understood, accumulating evidence indicates a key role of the peritumoral microenvironment. Brain cancer cells interact with neurons by forming synapses with them and by releasing exosomes, cytokines, and other small molecules. Strong interactions among neurons often lead to the synchronization of their activity. In this paper, we used an in vitro model to investigate the role of exosomes released by glioma cell lines and by patient-derived glioma stem cells (GSCs). The addition of exosomes released by U87 glioma cells to neuronal cultures at day in vitro (DIV) 4, when neurons are not yet synchronous, induces synchronization. At DIV 7–12 neurons become highly synchronous, and the addition of the same exosomes disrupts synchrony. By combining Ca^2+^ imaging, electrical recordings from single neurons with patch-clamp electrodes, substrate-integrated microelectrode arrays, and immunohistochemistry, we show that synchronization and de-synchronization are caused by the combined effect of (i) the formation of new neuronal branches, associated with a higher expression of Arp3, (ii) the modification of synaptic efficiency, and (iii) a direct action of exosomes on the electrical properties of neurons, more evident at DIV 7–12 when the threshold for spike initiation is significantly reduced. At DIV 7–12 exosomes also selectively boost glutamatergic signaling by increasing the number of excitatory synapses. Remarkably, de-synchronization was also observed with exosomes released by glioma-associated stem cells (GASCs) from patients with low-grade glioma but not from patients with high-grade glioma, where a more variable outcome was observed. These results show that exosomes released from glioma modify the electrical properties of neuronal networks and that de-synchronization caused by exosomes from low-grade glioma can contribute to the neurological pathologies of patients with brain cancers.

## Introduction

Gliomas are brain tumors with a very poor prognosis [1]; affected patients can develop epileptic discharges and a decline in cognitive functions [2–4]. These pathologies are caused by the tumoral infiltration of subcortical pathways [5, 6] but also by specific rearrangements of neuronal connections [2]. It is now established that neurons and glioma cells form synaptic connections that promote tumor growth and may exacerbate cognitive deficits [7–9].

Glioma stem cells (GSCs) have been identified within human brain tumors [10], while glioma-associated stem cells (GASCs) are representative of the tumor microenvironment of both high-grade (HGG) and low-grade glioma (LGG). Although not tumorigenic, GASCs support the aggressiveness of GSCs favoring their motility [11–13]. According to the WHO classification, HGGs comprise grades 3 and 4 gliomas, whereas LGGs comprise grades 2 gliomas [14].

Exosomes are 30–400 nm membrane vesicles with pleiotropic functions in intercellular communication [15]. Glioma-derived exosomes interact with the environment to favor tumor proliferation, invasiveness, angiogenesis, and immunosuppression [16].

The mechanisms underlying tumor-related epileptogenesis are still incompletely understood and include increases in extracellular glutamate [17, 18], cerebral edema, local metabolic imbalances, pH alteration, changes in neuronal and glial protein expression, and altered immunological activity [19, 20]. Moreover, glioma-derived exosomes are known to affect neural differentiation [21], thus potentially further contributing to the alteration of synaptic activity. However, the direct effect of these exosomes on synaptic physiology has not been yet described. Synchronization of the electrical activity among interacting neurons has been observed in a multitude of preparations, both in vitro and in vivo [22–25]. Synchronization among neurons, pacing cells and in general, among coupled oscillators represents a fundamental dynamical state that pervades biological systems [26]. Different theoretical approaches have been proposed to capture the nature of this collective phase transition [27–29] and its theoretical foundations from free energy minimization [30]. Among the most studied models of synchronization, we mention the integrate and fire model and its variations [28, 31, 32], the Kuramoto model [27], and models of coupled identical oscillators [29].

In the present manuscript, we (i) performed co-cultures of primary neurons and the U87 glioma cell line, and (ii) exposed primary neuron cultures to exosomes derived from U87 cells and from patient-derived GSCs. Both exosomes induced synchronization and de-synchronization depending on the state of the neuronal network: at day in vitro (DIV) 4 after dissociation neuronal cultures are not yet synchronous and the addition of exosomes and of glioma induce synchrony. In contrast at DIV 7–12, when neurons have achieved synchrony, the addition of glioma or exosomes disrupts synchrony. Our experimental results and modeling suggest that a lower threshold for spike initiation greatly contributes to the disruption of synchrony. We also tested the effect of the addition of exosomes from patients: exosomes from LGG patients disrupted synchrony, in contrast, the action of exosomes from HGG patients was more variable.

These observations show the complexity of the interactions between glioma and healthy neurons and provide additional cues to understand tumor-related epilepsy (TRE) and the cognitive and functional pathologies of patients affected by brain tumors.

## Results

### The dynamics of neuron-glioma co-cultures

We added U87 cells to 3 DIV neural cultures and followed them by live cell imaging of these co-cultures. Glioma cells were stably transfected with mCherry to distinguish them from the surrounding glia and neurons [33]. Glioma cells replicated vigorously (compare 0 and 94 h, Fig. S1A) and moved constantly; on some occasions, they appeared to drag a neuron or a glia cell during their motion (Fig. S1B). Under our experimental conditions, the rate of replication of glioma U87 was 0.026 ± 0.008 h^−1^ and the mean motility was 0.3 ± 0.01 μm/min; for GSCs: 0.031 ± 0.012 h^−1^ and 0.23 ± 0.01 μm/min (Fig. S1C–F).

### Glioma cells-derived exosomes induce network synchrony in young primary neuron cultures

**Fig. 1:**
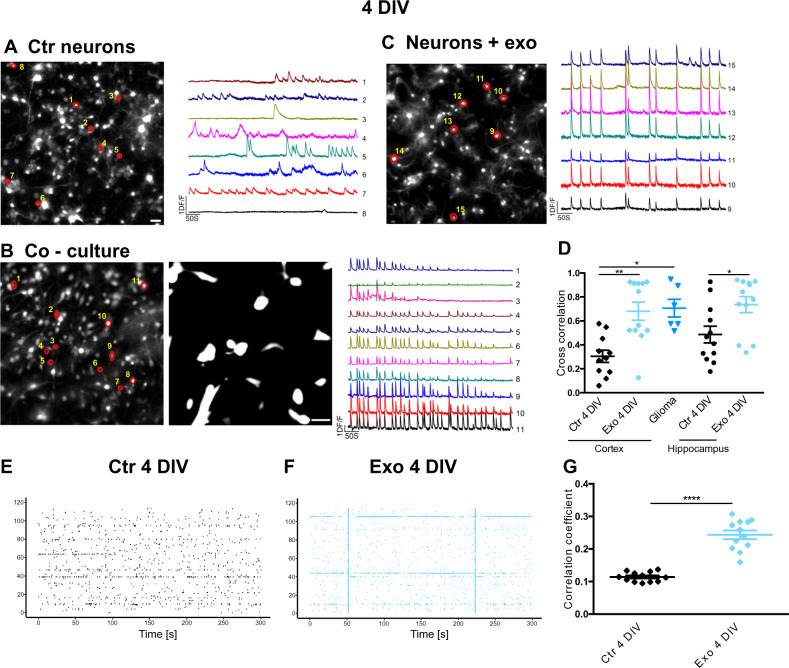
Glioma cell-derived exosomes induce network synchrony in young (DIV 4) primary neuron cultures. **A–C** Representative images of Fluo-4 loaded 3 DIV control cortical neuron cultures (**A**), of cultures co-cultured for 1 day with U87, mCherry expressing, glioma cells (**B**), and of cultures exposed for 1 day to U87-derived exosomes (**C**). ROIs indicate cells from which the representative Ca^2+^ traces are extracted. Scale bars, 50 µm. **D** Cross-correlation values of the various experimental groups. Data are shown as average ± standard error; statistically significant differences were assessed with the Wilcoxon–Mann–Whitney *U* test (to compare two groups) or Kruskal–Wallis test followed by Dunn’s multiple comparison post hoc test (to compare more than two groups); **p < 0.01, ***p < 0.001; *n* = 3 independent cell preparations, four coverslips for each preparation were analyzed and reported as superimposed individual points. **E** Representative raster plots of spike times simultaneously detected at 120 spatial locations (channels) by substrate-integrated MEAs: channels’ numbers are indicated on the *y*-axis. The spiking activity is displayed over a representative time window of 5 min, preceding the exposure to U87 exosomes (Ctr). **F** Representative recording of the same neuronal culture in (**E**) after 24 h of exosome exposure (Exo): the occurrence of network bursts is visible in the raster plot as vertical “stripes” spanning the entire MEA surface. Bursts are representative of the degree of synchrony of the network. **G** Pearson correlation coefficients among spike times from different channels over 15 min recordings. The average correlation values across 10 channels were analyzed and their distributions were compared under control conditions and after exosome, exposure using the Wilcoxon–Mann–Whitney test; ***p < 0.001; *n* = 12 MEAs, an average of 10 channels for each MEA.

Dissociated cortical and hippocampal neurons at DIV 2–4 display sparse and asynchronous spontaneous electrical activity (Fig. 1A). We added U87 cells to DIV 3 cultures and after one day of co-culture performed live Ca^2+^ imaging by adding Fluo-4, following Ca^2+^ activity in both neurons and U87 cells (Fig. S2). Spontaneous Ca^2+^ waves in a glioma cell (indicated by the broken vertical lines in Fig. S2C) were often followed by increased firing in neighboring neurons. In some cases (i.e., trace 5 in Fig. S2C), neurons within about 100–200 μm showed significantly increased electrical activity. Larger Ca^2+^ waves in glioma could elicit a sustained electrical activity in neighboring neurons, sometimes inducing a substantial elevation of intracellular Ca^2+^ (i.e., traces 6–8). The delay between the peak of the Ca^2+^ wave in the glioma cell and the increased electrical activity in neurons varied between 2 and 10 s and had a mean of 5.5 ± 1.8 s (*n* = 15).

Young neurons co-cultured with glioma cells displayed markedly synchronous Ca^2+^ signals compared to control cultures (Fig. 1A, B) and we asked whether this could be mediated by glioma-derived exosomes. To answer this question, we isolated exosomes from the U87 cell culture medium and added them to DIV 3 cortical cultures. We added approximately 30 μg/ml exosomes (4.2 × 10^3^ particles per cell), corresponding to 1–4 times the concentration in the conditioned medium, and cultured the network for an additional day. Interestingly, the addition of exosomes induced synchrony in the neuronal culture (Fig. 1C). The values of cross-correlation of Ca^2+^ transients under the various experimental conditions are shown in Fig. 1D; of note, exosomes had the same effect on synchrony also on hippocampal neuron cultures. We subsequently cultured cortical neurons for 3–4 days on multi-electrode arrays (MEAs) and added exosomes after recording basal spontaneous electrical activity. Under control conditions, only a sparse and poorly correlated activity was observed (Fig. 1E) but within 24 h after the addition of exosomes, some spike bursts appeared on most of the electrodes, which were highly correlated (Fig. 1F, G). The average correlation coefficient significantly increased from 0.11 to 0.24 (Fig. 1G).

Altogether, these data show that when neuronal cultures are not fully developed, the addition of U87-derived exosomes stimulates the establishment of synchrony, drastically modifying the properties of the network.

The size and number of exosomes in the samples were characterized by nanoparticle tracking analysis (NTA, Fig. S3A, B): the most concentrated particles had a dimension of 130 nm [34]. Atomic Force Microscopy (AFM) was applied to visualize vesicle heights and diameters, showing nanoparticles with a mean diameter ± sd = 100 ± 50 nm, confirming the NTA results. Few particles were also observed in the control sample, with a significantly smaller size distribution, likely corresponding to protein aggregates or residues from the isolation procedure (Fig. S3C). To further prove the purity of our preparation, the exosomal markers Flotillin1, Tumor susceptibility gene 101 (TSG-101), and Programmed cell death 6 interacting protein (Alix) [35] were analyzed by western blotting in total U87 cell lysates (CL) and in purified exosomes (EXO) from U87 cells and GSCs. These markers were indeed specifically visualized in the exosomal preparation, while the Golgi marker GM130 was present only in the cell lysate (Fig. S3D). A slight immunoreactivity for the TSG-101 marker appears also in U87 CL, as expected since this protein is expressed also intracellularly. Even though we cannot exclude the presence of some vesicles of other origins within the observed range of sizes, for the sake of simplicity we will refer to our vesicles as *exosomes* [36], since they represent the vast majority of our preparation as demonstrated by western blot analysis.

Altogether, these results confirm that the particles visualized are exosomes, excluding the presence of cellular contaminants.

### Glioma cells-derived exosomes disrupt network synchrony in mature primary neuron cultures

**Fig. 2:**
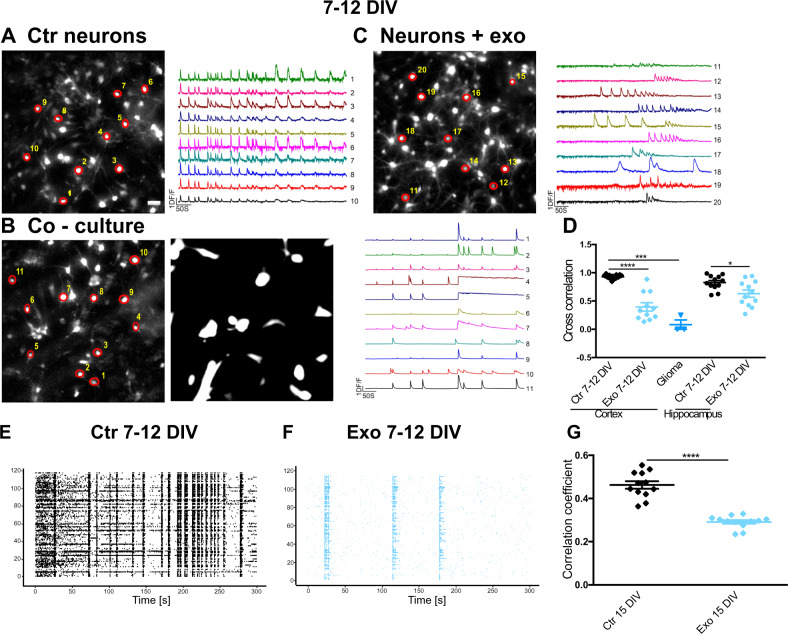
Glioma cells-derived exosomes disrupt network synchrony in mature (DIV 7–12) primary neuron cultures. **A–C** Representative images of a Fluo-4 loaded DIV 10 control cortical neuron culture (**A**), of neurons co-cultured for 1 day with U87, mCherry expressing, glioma cells (**B**), and of neurons exposed for 1 day to U87-derived exosomes (**C**). ROIs indicate cells from which the representative Ca^2+^ traces are extracted. Scale bars, 50 µm. **D** Cross-correlation values of the various experimental groups. Data are shown as average ± standard error, significant differences were assessed with Wilcoxon–Mann–Whitney *U* test (to compare two groups) or Kruskal-Wallis test followed by Dunn’s multiple comparison post hoc test (to compare more than two groups); *p < 0.05; ***p < 0.001; ****p < 0.0001; *n* = 3 independent cell preparations, four coverslips for each preparation were analyzed and reported as superimposed individual points. **E** Representative raster plots of spike times, simultaneously detected at 120 spatial channels by MEAs: channels’ numbers are indicated on the *y*-axis. The spiking activity is displayed over a representative time window of 5 min, preceding the exposure to U87 exosomes (Ctr). At this stage of development (DIV 7–12), network bursts are normally observed. **F** Representative recording of the same neuronal culture in **E** after 24 h exosome exposure (Exo), showing a lower occurrence of network bursts. **G** Pearson correlation coefficients among spike times in different channels over 15 min recordings. The average correlation values across 10 channels were analyzed and their distributions were compared under control conditions and after exosomes, exposure using the Wilcoxon–Mann–Whitney *U* test; ***p < 0.001; *n* = 12, an average of 10 channels.

Hippocampal and cortical neurons show a synchronous electrical activity at DIV 7–12 (Fig. 2A). In co-cultures of neurons and U87 cells, synchrony was disrupted. Bursts were not synchronous in sharp contrast with what was observed in the same cultures but in the absence of glioma (Fig. 2B). We exposed DIV 7–12 neuronal networks to U87-derived exosomes and we observed disruption of synchrony (Fig. 2C). Analysis of the cross-correlation matrices shows that most entries in control conditions are above 0.8 while following exosome treatment and in the presence of glioma cells values often fall below 0.4. Therefore, neuronal networks either co-cultured with glioma or exposed to U87 exosomes lost their synchrony (Fig. 2D); as noted for early-stage cultures, exosomes disrupted synchrony also on mature hippocampal neuron cultures. We repeated these experiments on DIV 7–12 cortical neuron cultures grown on MEAs and exposed to U87 exosomes for 24 h (Fig. 2E, F). Under these experimental conditions, neural networks displayed reduced synchrony.

Altogether, these data show that when glioma cells-derived exosomes interact with mature, synchronous neuronal cultures, synchronous activity is disrupted.

### Glioma cells-derived exosomes increase neuronal excitability

**Fig. 3:**
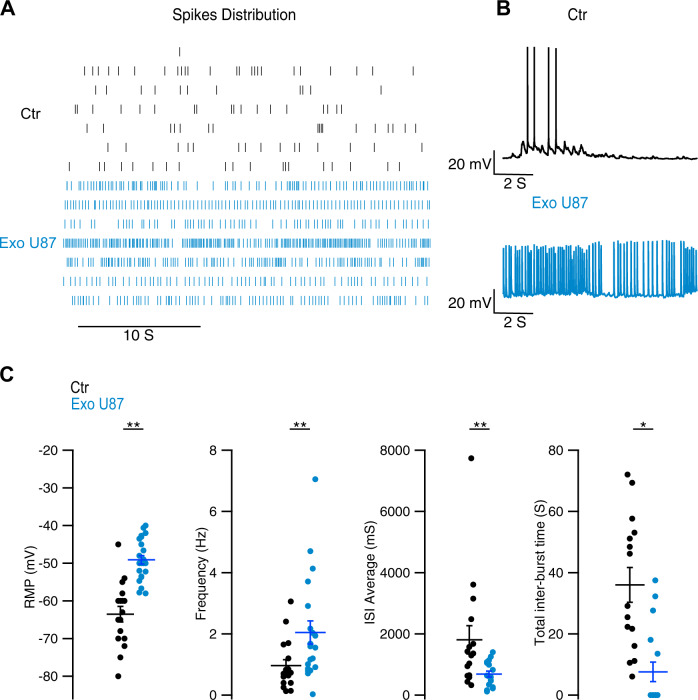
Glioma cells-derived exosomes increase spontaneous and evoked neuronal excitability. Spontaneous activity of hippocampal neurons was recorded in current clamp for 4 min in control neurons (ctrl) and in cultures treated for 24 h with U87-derived exosomes (U87). **A** Representative pattern of activity in a 30 s window is shown as a scatter plot (Black: Control *n* = 7, Blue: U87 treated *n* = 7) and **B** representative traces of single neurons in 10 s. Exosome-treated cells show a dense spiking activity that was absent in control conditions. **C** Exosome-treated neurons showed a striking switch of RMP to positive values and the induction of a long train of action potentials upon current injection. *Resting membrane potential (RMP)* was measured in the current clamp modality (current *I* = 0). Control: −63.529 ± 2.074 mV, *n* = 17 cells; U87 treatment: −49.083 ± 1.123 mV, *n* = 23 cells. *Action potential frequency*. Control: 0.9620 ± 0.197 Hz, *n* = 17 cells; U87 treatment: 2.046 ± 0.380 Hz, *n* = 23 cells. *Average inter-spike-interval (ISI)*. Control: 1806.49 ± 461.01 ms, *n* = 16 cells; U87 treatment: 683.68 ± 99.169 ms, *n* = 17 cells. *Total inter-burst time*. Control: 36.013 ± 5.678 s, *n* = 16 cells; U87 treatment: 7.586 ± 3.174 s, *n* = 17 cells. Data are shown as average ± standard error, significant differences were assessed with the Wilcoxon–Mann–Whitney *U* test. **p < 0.01; *p < 0.05.

We performed electrophysiological experiments on U87 exosome-treated hippocampal neurons at DIV 7–12 and found a much more intense spontaneous electrical activity in neurons exposed to exosomes (Fig. 3A, B). DIV 4 neurons were immature and showed a more depolarized RMP and no spontaneous activity (Fig. S4). Raster plots of the spontaneous activity of 7–12 DIV control neurons (Fig. 3A, black traces) show a variety of firing rates. In contrast, neurons exposed to exosomes had a much more vigorous spontaneous firing (Fig. 3A, light blue traces). This increased spontaneous firing was not caused by a difference in membrane capacitance or input resistance (Fig. S5) but by a more depolarized resting membrane potential (Fig. 3C) associated with a significant increase in spontaneous firing rate from a mean value of 0.96 ± 0.81 Hz up to 2.05 ± 1.70 Hz following exosomes treatment. Upon current injection, exosome-treated neurons generated longer action potential trains and at a higher frequency (Fig. 3C). Importantly, exosomes derived from non-transformed human astrocytes did not change the physiological properties of the mature neurons (Fig. S6).

Altogether, these data show that glioma cells-derived exosomes sharply increases spontaneous and evoked activity in cultured hippocampal neurons.

### Glioma cells-derived exosomes increase connectivity in DIV 4 neuron cultures

**Fig. 4:**
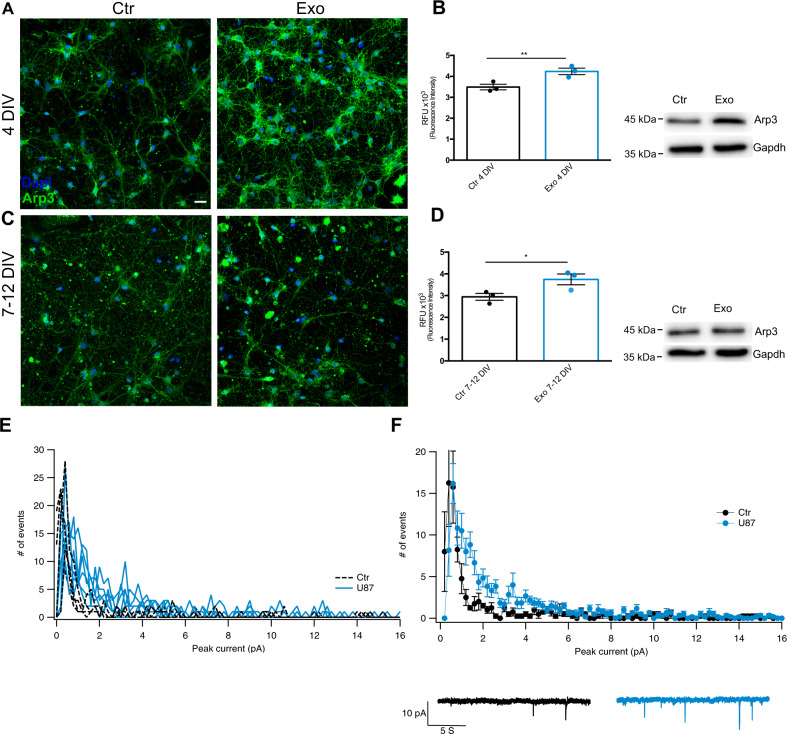
Arp3 expression is increased in hippocampal neurons after U87-derived exosome treatment. Representative confocal images of control (Ctr) and exosomes treated (Exo) hippocampal neuron cultures at 4 DIV (**A**) and 7-12 DIV (**C**) stained with antibodies for Arp3 (green channel) and DAPI for nuclei (blue channel). Scale bar: 20 µm. Arp3 staining quantification at 4 DIV (**B**) and 7–12 DIV (**D**) showing a statistically significant increase in both groups. Unpaired Student’s *t*-test, **p < 0.01, *n* = 3 independent cell preparations per group; quantification of 27 fields per group from three coverslips, was performed. Graphs show average ± SEM. Representative western blot images from the same experimental conditions are shown on the right; here, GAPDH is used to verify equal loading. **E** Comparison of synaptic currents at DIV 4. *Left*: overlay of peak currents distribution from several voltage-clamp recordings. Black: control neurons (*n* = 4); blue: U87 exosome-treated neurons (*n* = 6). Exosome treatment increased the number of synaptic events between 1 and 6 pA, suggesting higher connectivity. **F** average peak currents distribution from the recordings shown in (**E**). Kolmogorov–Smirnov test, **p < 0.01. Representative voltage-clamp traces at −70 mV in control and U87 exosome-treated hippocampal neurons are shown at the bottom.

Induction of synchrony at DIV 4 is likely caused by increased connectivity, which could originate from a higher number of synaptic contacts and/or increased synaptic efficacy. Genomic screening of the content of glioma-derived exosomes shows an abundance of the Arp2/3 complex [37], which promotes the branching of actin filaments and in cancer cells promotes invadopodia formation [38]. We found a higher Arp3 expression in neuronal cultures both at DIV 3–4 and DIV 7–12 by histochemistry and western blot analysis (Fig. 4A–D).

**Fig. 5:**
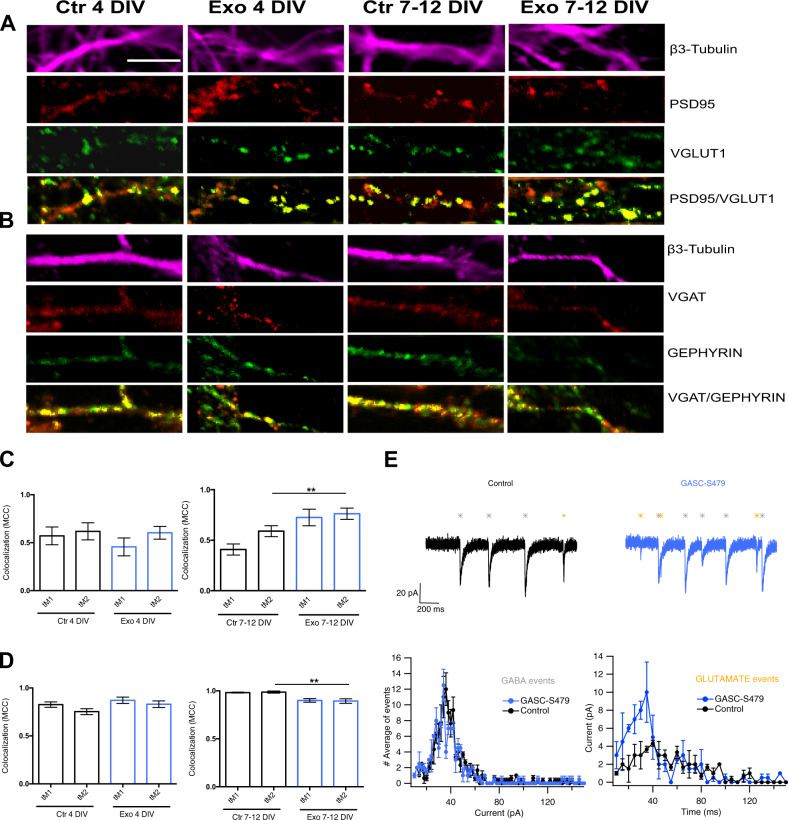
U87 exosomes increase the number of excitatory synapses and decrease inhibitory synapses at DIV 7–12 in hippocampal neurons. **A** Representative confocal images of excitatory synapses of hippocampal neurons from control (Ctr) and U87 exosome-treated (Exo) groups at DIV 4 and DIV 7–12. Neurons were identified by β3-tubulin staining (purple channel), and excitatory synapses were identified using double immunostaining with VGLUT1 (green channel)/PSD95 (red channel). Colocalization of pre- and post-synaptic markers is highlighted in yellow and corresponds to bona fide excitatory synapses. **C** VGLUT1/PSD95 colocalization was evaluated using Mander’s colocalization coefficient (MCC). tM1 corresponds to the fraction of VGLUT1 in compartments containing PSD95, while tM2 to the fraction of PSD95 in compartments containing VGLUT1. **B** Representative confocal images of inhibitory synapses identified by double immunostaining with VGAT (pre-synaptic, red channel)/GEPHYRIN (post-synaptic, green channel) under the same experimental conditions shown in (**A**). **D** VGAT/GEPHYRIN colocalization analysis was obtained as described in (**B**). Scale bar: 5 μm. Bar graphs show the average ± SEM of four different coverslips prepared from *n* = 4 distinct preparations. Unpaired Student’s *t*-test, **p < 0.01. For each coverslip at least 10 dendrites were analyzed. **E** *Upper panels*: Representative traces from recordings in voltage-clamp (Black: Control, blue: GASC-479 exosome-treated) showing spontaneous synaptic events evoked by GABA or glutamate (Gray and orange color, respectively). *Lower panels*: Average peak currents distribution from the recordings at DIV 7–12 for control cells (*n* = 3) and GASC-479 exosome-treated cells (*n* = 4) in cultures with low synaptic activity. *Left*: GABAergic events distribution, *Right*: Glutamatergic events distribution (*p < 0.05 Kolmogorov–Smirnov test).

To verify whether the higher expression of cytoskeletal proteins leads to increased connectivity, we performed voltage-clamp experiments in control neurons and in neurons treated with U87 exosomes and quantified the number of synaptic inputs converging on the impaled neurons. Under control conditions, DIV 4 neurons had modest synaptic inputs—in agreement with the lack of synchrony—but after exosome treatment, more frequent synaptic inputs were observed (Fig. 4E, F). Different results were obtained on DIV 7–12 synchronous cultures, as we will see in a later section (Fig. 5E).

Altogether, these data indicate that exposure to exosomes induces an increase in connectivity in DIV 4 cultures.

### Glioma cells-derived exosomes increase excitatory transmission in DIV 7–12 cultures

To investigate the mechanisms underlying the disruption of synchrony in mature cultures, we stained exosome-treated and control neurons with pre- and post-synaptic markers for excitatory and inhibitory synapses. We used VGLUT1 and PSD95 for the pre- and post-synaptic elements of glutamatergic synapses, VGAT, and Gephyrin to mark the pre- and post-synaptic elements of inhibitory synapses, respectively. In addition, tubulin β-III (TUBB3) was used to mark the neuronal cytoskeleton [39]. In Fig. 5A, representative images of the immunostaining of excitatory synapses are shown for all the experimental conditions. After exosome treatment at DIV 4, PSD 95 (in red) and VGLUT1 (in green) co-localization (yellow points) is not significantly changed, while at DIV 7–12, co-localization of pre and post-synaptic elements is significantly higher in exosome-treated neurons than in control cells (Fig. 5C). In Fig. 5B, D images and graphs representing the co-localization of inhibitory synapse proteins are shown. At DIV 4, there are no differences in the number of inhibitory synapses between the exosome-treated and control group, while at DIV 7–12 we observe a significantly decreased co-localization in the exosome-treated group compared to the control.

Current clamp recordings showed that exosome exposure induced neurons to fire longer action potential trains and at a higher frequency (see Fig. 3). To evaluate the role of synaptic input in this phenomenon we performed voltage-clamp recordings at −70 mV (Fig. 5E, *upper traces*) to study synaptic currents in control neurons and in neurons exposed to exosomes. Of note, for these experiments, we used LGG patient-derived exosomes. The distribution of the amplitudes of detected synaptic currents (*n* = 23 and 31 neurons for control and exosome-treated groups, respectively) did not show any statistically significant difference. However, if we considered only experiments with low synaptic activity, where glutamatergic and GABAergic events can be easily distinguished by their time course (Fig. S7) [40, 41], we found—in two experiments with exosomes from a patient—a higher occurrence of glutamatergic events in exosome-treated cultures, in agreement with our histochemical analysis (Fig. 5E, *lower panel*).

### The effect of patient-derived exosomes on neural network activity

**Fig. 6:**
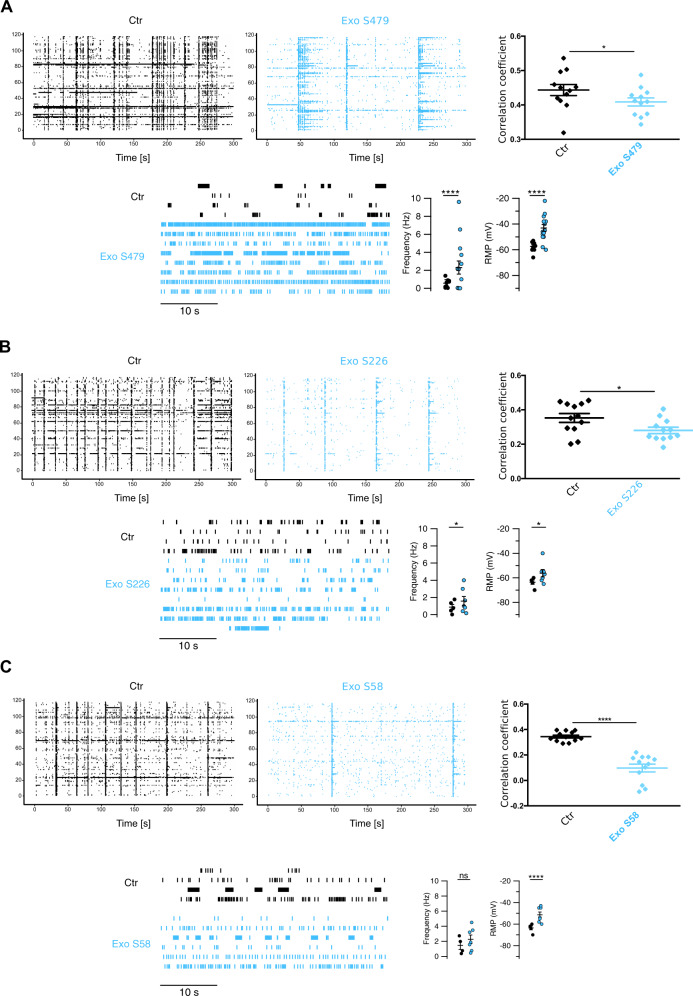
Exosomes derived from LGG patients increase neuronal excitability and disrupt synchrony. Network-wide and single-cell electrophysiological phenotype of neurons treated with exosomes from LGG patients (**A**: S479; **B**: S226; **C**: S58). Raster plots from MEA recordings and correlation coefficients among spike times are shown in the upper panels, before (Ctr, black) and 24 h after exosome incubation (Exo, blue). The *y* axes on the raster plots represent channels’ numbers. The average correlation values across 10 channels were calculated and their distribution compared using Wilcoxon–Mann test; *p < 0.05, ***p < 0.001; *n* = 12, average of 10 channels. In the lower panels, raster plots of single-cell activity are shown for neurons treated with patients’ exosomes and the respective controls. The distributions of firing rate (Hz) and resting membrane potentials (RMP) are shown; statistics were performed using the Wilcoxon–Mann–Whitney *U* test; *p < 0.01, ****p < 0.0001; *n* = 9/15 for S479, 5/7 for S226, 4/7 for S58 (neurons analyzed in Ctr/Exo samples, respectively).

**Fig. 7:**
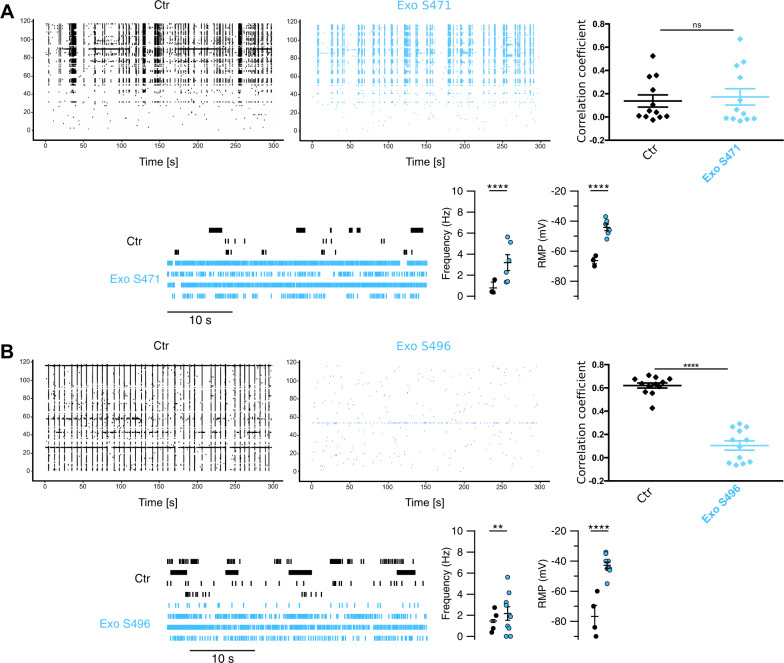
Exosomes derived from HGG patients have variable effects on neuronal excitability and synchrony. Network-wide and single-cell electrophysiological phenotype of neurons treated with exosomes from HGG patients (**A**: S471; **B**: S496). Raster plots from MEA recordings and correlation coefficients among spike times are shown in the upper panels, before (Ctr, black) and 24 h after exosome incubation (Exo, blue). The *y* axes on the raster plots represent channels’ numbers. The average correlation values across 10 channels were calculated and their distribution compared using Wilcoxon–Mann–Whitney *U* test; **p < 0.001, ****p < 0.0001; *n* = 12, average of 10 channels. In the lower panels, raster plots of single-cell neuronal activity are shown for neurons treated with patients’ exosomes and the respective controls. The distributions of firing rate (Hz) and resting membrane potentials (RMP) are shown; statistics were performed using the Wilcoxon–Mann–Whitney *U* test; **p < 0.001, ****p < 0.0001; *n* = 3/6 for S471, 4/9 for S496 (neurons analyzed in Ctr/Exo samples, respectively).

To bring our findings closer to the clinical setting, we tested the effect of exosomes derived from three LGG patients (S479, S226, and S58, Fig. 6) and two HGG patients (S471 and S496, Fig. 7). Given the paucity of the available material from these patients, the number of independent experiments was limited (*n* = 1–2 for MEA recordings; *n* > 8 for intracellular recordings from at least five different dishes per patient). MEA recordings in DIV 7–12 cortical neurons show spontaneous synchronous firing (Fig. 6A–C, black traces, *upper left panels*). However, following 24 h incubation with exosomes from LGG patients, spiking synchronicity was disrupted (Fig. 6A–C, blue traces, *upper middle panels*): the correlation coefficient among spike times was significantly decreased in all three cases (Fig. 6A–C, *upper right graphs*). These results are consistent with the ones obtained from U87 exosomes (Fig. 2). In the case of exosomes derived from patient S479, we observed large waves of electrical activity invading the entire network: these ‘superbursts’ appeared to be the collective expression at the network level of the pro-epileptic behavior induced by exosomes at the single cell level (see Fig. 3).

Given the lower heterogeneity of hippocampal neurons in culture compared to that of cortical neurons [42, 43], we performed intracellular recordings on hippocampal neurons treated under the same experimental conditions. Neurons treated with exosomes from LGG patients exhibited a more depolarized resting membrane potential (RMP) and episodes of vigorous spontaneous firing (Fig. 6A–C*bottom panels*).

Exosomes derived from HGG patients gave the following results: in the case of patient S471, the spike synchronicity and spontaneous firing were maintained after exosome incubation. Similarly, intracellular recordings showed a more vigorous spontaneous firing in some neurons, while other neurons had a firing very similar to controls (Fig. 7A). In contrast, incubation with exosomes from patient S496 depressed the electrical activity and several electrodes became silent (Fig. 7B). At the intracellular level, several neurons treated with exosomes from patient S496 fired continuously because of the depolarization of the resting membrane potential, but in other neurons, this depolarization was so large that neurons did not show any spontaneous activity, presumably for the inactivation of the Na^+^ current induced by the excessive depolarization. These “silent” neurons may explain the low electrical activity observed with MEAs (Fig. 7B).

## Discussion

In this manuscript, we studied the impact of U87 cells- and glioma-derived exosomes on the activity of young and mature primary neurons. The addition of glioma cells or glioma-derived exosomes to young neuronal cultures stimulates the establishment of synchrony. In contrast, when mature neurons come in contact with glioma cells or exosomes synchronous firing is disrupted, in some cases exhibiting a super bursting activity in which most of the neurons enter in a reverberating pro-epileptic activity lasting several seconds. Similar results were obtained with exosomes from LGG patients, while more variable results were obtained with exosomes from HGG patients.

Synchronization depends on the culture density as well as on the physical and biochemical environment, such as substrate stiffness [44], and less critically on the nature of cultured neurons [45]. The number and efficiency of synaptic connections, the degree of coupling, the relative weight of inputs from other neurons, and the intrinsic firing of each neuron are all critical parameters. In vitro studies identified numerous players for the establishment and maintenance of synchronous firing, including AMPA and NMDA receptors [46], nicotinic acetylcholine receptors [47], and voltage-gated Ca^2+^ channels [48], and many others. The integrate and fire (IF) model [28, 31] provides a theoretical perspective to understand synchronicity and its disruption [32] and will be used later to verify some mechanisms. On the basis of our experimental results we propose that (i) in young cultures, the main mechanism underlying exosome-induced synchronization is an increase of coupling and connectivity, likely through Arp2/3 overexpression, while (ii) in mature cultures the main mechanism underlying the disruption of synchronicity is the decrease of the firing threshold, accompanied by additional changes in synaptic properties.

Extracellular vesicles and exosomes contain a vast repertoire of bioactive molecules including cytokines, proteins, lipids, and RNA segments [49], all of which can target recipient cells and modify synaptic properties in subtle ways. Amongst these proteins, we focused on Arp2/3, an important modulator of the neuronal cytoskeleton, whose expression increased in both young and mature neurons treated with exosomes. A recent proteomic analysis of glioblastoma-derived extracellular vesicles identified proteins involved in filopodia formation, including Arp3, in exosomes derived from the most aggressive tumors [37]. The increased Arp3 levels in exosome-treated cultures are at this moment only a correlational observation. The increased amount of Arp3 proteins observed in neurons could be directly delivered from exosomes and/or can be induced indirectly through stimulation of intracellular pathways. We speculate that, in young cultures, higher levels of Arp2/3 may initiate the process of synaptogenesis, promoting the formation of filopodia and contacts between neurons, while in mature cultures Arp2/3 may contribute to the maintenance and/or stabilization of excitatory boutons (Figs. 4A–D and 5). Indeed, our electrophysiological measures on immature neurons show a higher number of synaptic events (Fig. 4D, E), further supporting the idea of accelerated synaptogenesis in exosome-treated DIV 3–4 cultures. In mature cultures, instead, exosome-treated neurons are characterized by a more depolarized resting membrane potential (Fig. 3), which leads to increased excitability. Interestingly, exosomes derived from non-transformed human astrocytes do not have the same effect as glioma-derived exosomes on network excitability (Fig. S6). Astrocyte-derived exosomes contain neurotrophic factors that promote neuronal survival, neurite outgrowth, and synaptic function [50]. On the other hand, glioma-derived exosomes contain a much wider array of molecules that stimulate cancer cell growth and invasion, neo-angiogenesis, oncogenic transformation, and suppression of the immune response [51]. Thus, the comparison of the content of the vesicles released by these two types of cells is not straightforward. In our opinion, molecules that impact the immune system are particularly relevant. The mechanisms by which exosomes induce this phenotype are presently unknown but may include the modulation of Na^+^ conductance directly through specific miRNAs or indirectly by acting on other membrane receptors. Indeed, our future studies will aim at identifying these mechanism(s) in more detail; they will be relevant to developing novel therapies targeting brain excitability induced by glioma, which—as mentioned before—often manifests itself as epilepsy [3].

**Fig. 8:**
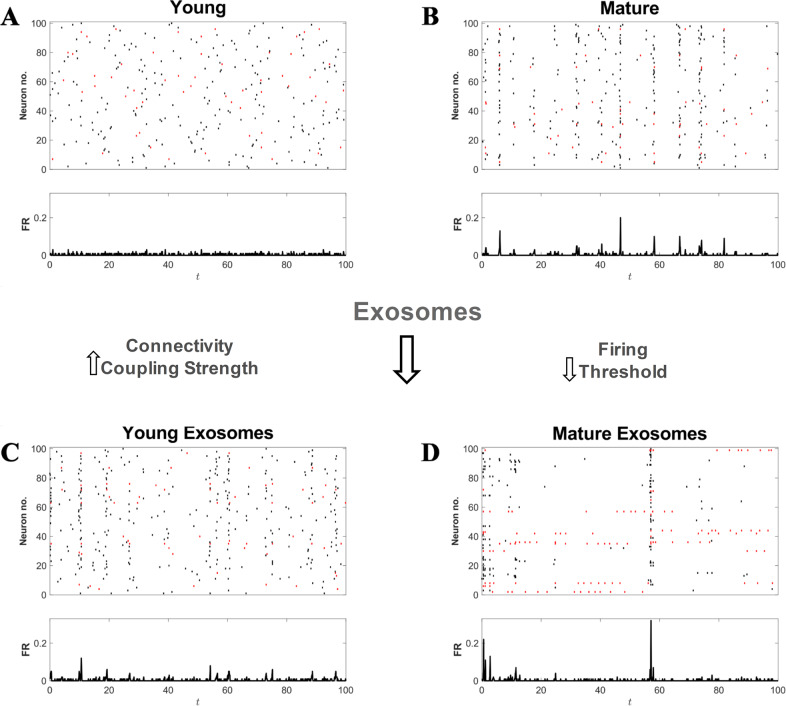
Mechanisms for synchrony and its disruption. The raster plots and the firing rate of in-silico neuronal cultures are plotted for young and mature cultures in the absence and in presence of exosomes. Young immature cultures (**A**) are modeled by a sparse network with low coupling, which is unable to produce consistent population spikes, whereas mature cultures are modeled by a network with strong coupling, leading to the emergence of population spikes (**B**). The presence of exosomes is modeled according to the experimental evidence. For young cultures, the presence of exosomes is modeled by an increase in connectivity and an increase in the strength of the synaptic coupling (**C**), which allows for the appearance of population spikes, as observed in our experiments. For mature cultures (**D**) the presence of exosomes is modeled by decreasing the neuronal threshold for firing. This leads to a firing-inhibition regime, with reduced firing and only rare population events. Due to the large coupling, population events may now recruit large portions of the network. The leaky integrate and fire model used for these in-silico realizations are described in detail in the SI where we provide the meaning and interpretation of the different parameters used in the simulations. The young culture in panel **A** is simulated by setting the model parameters to *d*_c_ = 0.25, *V*_th_ = 2.0 mV, *σ* = 10.0 pA and *I*_0_ = 6.0 pV. The presence of exosomes (panel **C**) in this case was modeled by increasing the connectivity (*d*_c_ = 0.25) and the coupling (*σ* = 130.0 pA). The mature culture in panel (**B**) is simulated by setting the model parameters to *d*_c_ = 0.5, *V*_th_ = 3.0 mV, *σ* = 180.0 pA and *I*_0_ = 10.0 pA. The presence of exosomes was modeled by *V*_th_ = 0.50 pA (panel **D**). All the panels are obtained from in-silico neuronal networks of *N* = 100 neurons.

We have performed an in-silico simulation of neuronal dynamics to study how exosomes affect synchronization. Neuronal dynamics were modeled by a simple leaky IF model including the detailed balance between the inhibitory and excitatory populations over a random geometric network (see Fig. S8 for details). The model illustrated in Fig. 8 reproduces our main experimental observations on synchrony and rupture of synchrony, indeed, synchrony is obtained by increasing the connectivity and synaptic efficacy, while the disruption of synchrony is obtained primarily by lowering the firing threshold.

The present manuscript is primarily based on the exosomes released by U87 cells, considered a useful model of glioma [33, 52]. We attempted, however, to perform similar experiments with exosomes obtained from patients and more precisely from exosomes released from GSCs and GASCs from the cerebral tissue excised during surgery. Therefore, the exosomes have not been directly obtained from the cerebral tissue. It is also important to underline that the properties of exosomes released from glioma located in different regions of the brain tumor are likely to be different. Having in mind all these caveats, some conclusions can be drawn from our results. Similar to what was observed with U87-derived exosomes, exosomes from our three LGG patients disrupt synchrony in mature neuronal cultures and increase the spontaneous firing of neurons (Fig. 6). It is important to underline that the effects observed on mature networks are the most relevant from the clinical perspective, as gliomas originate in the brain of adult patients, where neural circuits are mature and fully developed. In mature networks, the physiological level of synchrony is disrupted and replaced by sparse un-correlated firing interspersed by large episodes of intense firing, reminiscent of the epileptic events in patients with LGG [2]. Therefore, it is possible that, besides causing epileptic events, LGGs disturb the neuronal processing of healthy regions of the brain, which can be reached by the exosomes released by the brain tumor, thus impairing physiological cognitive functions.

For what concerns exosomes from patients with HGG, in one case the global electrical activity was only marginally affected but in the other case several neurons had a very depolarized membrane voltage potential and could not operate properly. More HGG samples have to be analyzed in order to draw general conclusions from these data. However, these results are interesting when considering the fact that HGG is less often associated with epileptic discharges [2].

## Conclusions

Overall, the present manuscript shows that glioma-derived exosomes have a profound effect on neuronal networks’ activity and synchrony. Thus, glioma cells not only form direct synaptic connections with neurons [7–9] but are also able to modify the properties of neurons and neuronal assemblies through the release of exosomes. These results, although obtained in vitro, suggest that brain tumor cells modify the electrical properties of neurons and of their synapses when they infiltrate into neuronal networks.

## Materials and methods

### Cell cultures

Primary neuron cultures were prepared from Wistar rats (P0–P2), according to the guidelines of the Italian Animal Welfare Act and the European Union guidelines for animal care (d.1.116/92; 86/609/C.E.). Their use was approved by the Local Veterinary Service, the SISSA Ethics Committee board, and the National Ministry of Health. Both hippocampal and cortical neurons were used for the experiments. Hippocampus and cortex isolated from the brain were subjected to enzymatic digestion with a solution containing NaCl 136.9 mM, KCl 4.9 mM, Na_2_HPO_4_ 7 mM, 4-(2-hydroxyethyl)-1-piperazineethanesulfonic acid (HEPES) 25.2 mM, NaHCO_3_ 4.2 mM, Kinurenic Acid 200 μM and D(-)-2-Amino-5-phosphonopentanoic acid (APV) 25 μM (all from Sigma-Aldrich^®^, St. Louis, MO). Neurons obtained from hippocampus and cortex digestion were then plated on 15 mm glass coverslips (Menzel-Glaser, cat. no. CB00150RA1) previously coated with Poly-l-Ornithine (Sigma-Aldrich^®^, cat. no. P4957) at a concentration of 0.5 μg/ml and cultured in Neural Basal-A Medium (Thermo Fisher Scientific, USA, Cat. no. 21103049) supplemented with GlutaMAX^TM^ Supplement (Thermo Fisher Scientific, cat. no. 35050061), Gentamicin solution 10 mg/mL (Sigma-Aldrich^®^, Cat. no. G1272) and B27^TM^ Supplement (Thermo Fisher Scientific, cat. no. 17504044) at 37 °C, 5% CO_2_.

Human Astrocytes (HAs) (Thermo Fisher Scientific, cat. no. N7805100) were cultured in Dulbecco’s modified Eagle medium (DMEM) with GlutaMAX™ supplemented with 10% fetal bovine serum (FBS) (Invitrogen, Life Technologies, Gaithersburg, MD, cat. no. 31966047 and ECS0180L), 1% PenStrep (100 U/ml penicillin and 100 μg/ml streptomycin, Thermo Fisher Scientific, cat. no. 15070063) and N2 Supplement 100X (Thermo Fisher Scientific, cat. no. 17502048). Once 70–80% of confluence was achieved, the cells were re-plated. Cultures were maintained at 37 °C, 5% CO_2_ and the medium was replaced every 3 days. HA-derived exosomes were used as a control to verify the specificity of the effect of the U87 exosomes.

Human U87 GBM cells (Sigma-Aldrich^®^, cat. no. 89081402) is a cell line with epithelial morphology, originally isolated from a malignant glioma of a male patient. U87 cells were cultured in DMEM with GlutaMAX™ (Thermo Fisher Scientific) supplemented with 10% FBS (Invitrogen, Life Technologies), 1% PenStrep (100 U/ml penicillin and 100 μg/mL streptomycin, Thermo Fisher Scientific). The medium was replaced every 3 days. Once 70–80% of confluence was reached, cells were re-plated.

To obtain glioma-associated stem cells (GASCs), human GBM samples were collected by the Neurosurgery Department of the Azienda Ospedaliera Universitaria of Udine. The local Ethics Committee, Comitato Etico Unico Regionale del Friuli Venezia Giulia, approved this investigation (protocol number 718345, opinion 196/2014/Em). Briefly, tissue samples were mechanically/enzymatically dissociated and single-cell suspensions were cultured in adhesion on fibronectin-coated dishes as previously described [12]. All cells were routinely tested for mycoplasma contamination.

### Exosome isolation

When cells reached 70–80% confluence, FBS was substituted with 1% exosome-depleted serum (Thermo Fisher Scientific, cat. no. A2720803) is contained in the culture medium; cells were then left in culture for an additional 24 h (HA and U87 cells) or 48 h (GASCs), before collecting media to perform exosome isolation. After centrifugation at 2000 × *g* for 30 min to remove cells and debris, the supernatant was transferred into new tubes and subjected to exosome isolation using Total Exosome Isolation reagent (Invitrogen, Life Technologies, cat. no. 4478359) following the manufacturer’s protocol. Briefly, 0.5 volumes of the reagent were added to the supernatant and the solution was incubated overnight at 4 °C. After ultra-centrifugation at 10,000 × *g* for 1 h at 4 °C, the supernatant was removed and the exosome pellet resuspended in 150 μl of PBS and stored at −80 °C. To treat control samples, each type of culture medium used for incubation to isolate exosomes, which was never been in contact with cells, was subjected to the same protocol of exosome extraction. Cortical and hippocampal neurons were treated using 4.2 × 10^3^ particles (exosomes) per cell for 24 h at two different stages of maturation: 4 DIV and 7–12 DIV.

### Exosome characterization

#### NanoSight

Isolated exosomes were analyzed using a 405 nm (violet) laser at the NanoSight LM10 (Malvern Panalytical) to verify their size and concentration.

#### Atomic force microscopy

Atomic Force Microscopy (AFM) measures were performed to visualize and further characterize exosomes. AFM images were acquired using a commercially available microscope (MFP-3D Stand Alone AFM from Asylum Research, Santa Barbara, CA) in dynamic AC-mode in liquid using commercially available silicon cantilevers (BL-AC40TS- C2, Olympus Micro Cantilevers, nominal spring constant 0.09 N m ^−1^ and resonant frequency 110 kHz). For the preparation of the sample: a freshly cleaved muscovite mica sheet (Ruby Muscovite Mica Scratch Free Grade V-1, Nanoandmore GMBH, USA) was incubated with a drop of poly-l-ornithine solution 0.01% (Sigma-Aldrich) for 15 min at room temperature (RT). Subsequently, the excess poly-lysine was removed by performing five washes with Milli-Q water. A 15 µm drop of exosome suspension was then applied to the poly-ornithine-coated mica surface at RT for 15–30 min to allow the vesicles to bind the surface via electrostatic interactions. Samples were then washed with PBS 5 times and transferred to the AFM microscope. For each sample, 3–5 images with 10 × 10 µm of scan size and with a resolution of 1024 × 1024 pixels (pixel size ~10 × 10 nm) were acquired. The AFM images were analyzed with the Gwyddion^®^ software to extract vesicle heights and diameters.

#### Exosome markers expression

The expression of exosome markers was evaluated by Western Blot. Exosomes isolated from cell culture medium were resuspended in CHAPS Cell Extract Buffer (Cell Signaling Technology, Danvers, MA, USA cat. no. 9852S) supplemented with protease inhibitor cocktail (Thermo Fisher Scientific, cat. no. 78430). 4X Laemmli buffer was added to the samples and, after denaturation at 95 °C for 5 min, 30 µg/lane were loaded on 10% SDS-polyacrylamide gel. Proteins were transferred onto Hybond^®^ ECL^TM^ nitrocellulose membranes (Amersham Biosciences, cat. no. LC2000) by the semi-dry system Trans-Blot^®^ Turbo^TM^ Transfer System (Biorad, cat. no. 1704150) at 1.3 A and 25 V for 1 h. After blocking in 3% BSA (Bovine serum albumin, Sigma-Aldrich^®^) for 1 h at RT, membranes were incubated with the primary antibodies overnight at 4 °C with gentle shaking. Rabbit polyclonal anti-Flotillin 1 (Abcam, Cambridge, UK, 1:500, cat. no. ab41927), rabbit polyclonal anti-TSG101 (Abcam, 1:500, cat. no. ab133586), and mouse monoclonal anti-Alix (1:1000, Cell Signaling Technology, cat. no. 2171) were used as exosome markers, while rabbit monoclonal anti-GM130 (1:1000, Cell Signaling Technology, cat. no. 12480) was used as Golgi apparatus marker. After three 5 min washes in TBS with 0.1%Tween 20 (Sigma-Aldrich^®^, cat. n. P9416), membranes were incubated with goat anti-rabbit and anti-mouse HRP-conjugated secondary antibodies (Agilent, Palo Alto, CA, USA, cat. no. P0449 and P0447) for 1 h at RT, washed three times as previously, incubated for 3–5 min. in Immobilon^®^ ECL Ultra Western HRP Substrate (Sigma-Aldrich^®^, cat. no. 42029053) and analyzed with the transilluminator NineAlliance^TM^ (Uvitec, Cambridge, UK).

#### Calcium imaging

In order to perform Ca^2+^ imaging experiments and observe intracellular Ca^2+^ transients after exosome treatment, 1.0 × 10^5^ hippocampal or cortical neurons were cultured on 15 mm glass coverslips. On the day of the experiment, cells were first incubated at 37 °C for 20 min with 4 μM membrane-permeable Ca^2+^ dye Fluo-4 AM (Thermo Fisher Scientific, cat. no. F14201) and 0.1% (v/v) Pluronic F-127 (Thermo Fisher Scientific, cat. no. P3000MP) in Ringer’s solution (145 mM NaCl, 3 mM KCl, 1.5 mM CaCl_2_, 1 mM MgCl_2_, 10 mM glucose and 10 mM Hepes, pH 7.4); after three washes in Ringer’s solution for 20 min each, the coverslips were transferred to a 35 mm µ-Dish for high-end microscopy (Ibidi, Gräfelfing, Germany, cat. no. 81156). Recordings were performed at 37 °C for 10 min, using a Nikon Eclipse Ti-U inverted microscope equipped with a piezoelectric table (Nano-ZI Series 500 μm range, Mad City Labs), an HBO 103W/2 mercury short arc lamp (Osram, Munich, Germany), a mirror unit (465–495 nm excitation bandpass filter, 505 nm dichroic, 515–555 nm emission bandpass filter) and an Electron Multiplier CCD Camera C9100-13 (Hamamatsu Photonics, Japan). Images were acquired using the NIS Element software (Nikon, Japan) with a S-Fluor ×20/0.75 NA objective, at a sampling rate of 0.3 and with a spatial resolution of 256 × 256 pixels. Image analysis was performed using ImageJ software (Rasband, W.S., ImageJ, U.S. National Institutes of Health, Bethesda, MD, USA) and a custom-build MATLAB code (The MathWorks, Inc., Natick, MA, USA, https://www.mathworks.com, as previously described [53]. To avoid image saturation, excitation light intensity was attenuated by ND4 and ND8 neutral density filters (Nikon, Tokyo, Japan).

#### Extracellular electrophysiological recordings

Commercial MEAs (Multichannel Systems GmBH, Reutlingen, Germany) were used to monitor the extracellular electrical activity in cortical neuronal cultures. Each MEA contains 120 titanium nitrate (TiN) microelectrodes with a diameter of 30 μm and an inter-electrode distance of 100 μm, arranged as a 12 × 10 regular layout. MEA’s electrodes detected extracellular action potentials from neurons located in their proximity. We employed an electronic multichannel amplifier (MEA2100-Mini-120-System, Multichannel Systems GmBH, Reutlingen, Germany) with 10–10,000 Hz bandwidth and an amplification factor of 1. Recordings were performed inside of a (dry) incubator, at 37 °C and 5% CO_2_ (C150, Binder GmbH, Tuttlingen, Germany). Evaporation was strongly reduced by sealing MEAs with PDMS caps and controlled over 24 h recording sessions by direct measurements: changes in osmolality did not exceed 10% after 24 h. Extracellular raw electrical signals were sampled at 25 kHz/channel and digitized at 16 bits of resolution by the MEA2100–Mini USB interface. The raw voltage traces were analyzed with custom scripts written in Julia as previously reported [54], to extract the time of occurrence of action potentials at each MEA microelectrode. Briefly, for each recording channel, a threshold for peak detection was set adapted depending on the background electrical noise [55]. The Pearson correlation coefficients between spike times in different channels were computed and averaged across 10 s time windows, using Matlab codes.

#### Immunofluorescence staining

For immunofluorescence experiments, cells were plated at a density of 1 × 10^5^ on 15 mm glass coverslips and cultured for either 3 or 6–11 days, after which 4.2 × 10^3^ exosomes per cell were added for 24 h. Subsequently, primary cultures were rinsed three times in PBS and then fixed in 4% paraformaldehyde for 10 min at RT and kept at 4 °C until staining was performed. For the immunofluorescence staining, fixed cultures were rinsed three times in PBS to remove PFA, then a glycine solution (glycine 1 mM in PBS) was added for 5 min to reduce autofluorescence. To perform immunostaining of intracellular proteins an additional step with Triton^TM^ X-100 (Sigma-Aldrich^®^) (0.2% in PBS) of 5 min was added. Cells were then treated with a blocking solution, to saturate unspecific binding sites, composed of 10% Normal Goat Serum (Sigma-Aldrich^®^, cat. no. NS02L-1ML), 0.1% Tween^TM^ 20 Surfact-Amps^TM^ detergent solution (Thermo Fisher Scientific) and 5% BSA at RT for 45 min.

Incubation with the following primary antibodies: mouse monoclonal anti-Actin-related protein3 (Arp3; Abcam, 1:200, Cat. no. ab4967), polyclonal chicken anti-β3 tubulin (Abcam, 1:500, cat. no. ab41489), guinea pig polyclonal anti-vesicular glutamate transporter-1 (VGLUT1; Thermo Fisher Scientific, 1:2000, cat. no. AB 5905), polyclonal rabbit anti-postsynaptic density protein 95 (PSD95; Abcam, 1:500, cat. no. ab18258), mouse monoclonal anti-vesicular GABA transporter (VGAT; Synaptic Systems, 1:200, cat. no. 131011) and rabbit polyclonal anti-Gephyrin (Genetex, 1:250, cat. no. 109734) was conducted at 37 °C for 1 h. The following secondary antibodies were used for the detection: goat anti-mouse Alexa Fluor 488 (Invitrogen, Life Technologies, 1:600, cat. no. A11029), goat anti-rabbit Alexa Fluor 594 (Invitrogen, 1:600, Cat. no. A11037), donkey anti-chicken Biotin conjugated (Invitrogen, 1:600, Cat. no. SA1-72003); streptavidin Alexa Fluor 647 (Invitrogen, 1:250, Cat. no. S21374) was used for the detection of biotinylated secondary antibody.

For image acquisition, an inverted Nikon A1R confocal microscope (Nikon, Japan) was used. Images were acquired using the NIS Element Advanced Research Software (Nikon, Japan) with either a ×40/0.95 NA or a ×60/1.40 NA oil-immersion objective and a 3-fold zoom with a spatial resolution of 1024 × 1024 pixels. Quantification of fluorescence intensity was measured in regions of interest using the ImageJ software (NIH). Quantification of colocalization was measured with the ImageJ software, through the Coloc2 plug-in.

#### Intracellular electrophysiological recordings

Primary neurons were plated at a density of approximately 1 × 10^5^ on 15 mm glass coverslips and cultured for 6–11 days, after which 4.2 × 10^3^ particles per cell were added for 24 h. The electrophysiology experiments were performed in the whole cell patch clamp modality. Hippocampal neurons at 4 DIV were transferred to a recording chamber with a continuous Ringer’s perfusion, visualized in an inverted microscope Olympus IX70 equipped with ×20 and ×40 objectives. Neurons were identified by characteristic morphology. Patch pipettes were made of borosilicate glass (WPI, Sarasota, FL, USA) with a PP-830 puller (Narishige, Tokyo, Japan) and had a resistance of 3–5 MΩ. Voltage recording data were done with an Axopatch 1D amplifier controlled by Clampex 9 via a Digidata 1332A (Axon Instruments, Union City, CA, USA), low-pass filtered at 2 kHz, and sampled at 10 kHz.

Patch pipettes were filled with an intracellular solution contained (in mM) 140 KCl, 4 MgCl_2_, 10 HEDTA, and 10 HEPES, adjusted to pH 7.2 with KOH. Extracellular Ringer’s solution was composed of 140 NaCl, 5 KCl, 1 CaCl_2_, 1 MgCl_2_, 10 HEPES, and 10 mM glucose, pH 7.4. Patching pipettes were pulled from borosilicate capillaries (WPI) with a Narishige PC-10 puller and had resistances of 5–10 MΩ. Electrophysiology recordings were obtained using an Axoclamp 700b amplifier controlled by Clampex 10.6 via a Digidata 1550B (Molecular Devices) and filtering with 5 kHz low pass. Recordings in the current clamp were performed in current equal 0 or follower voltage mode. Cells in voltage clamp experiments had a holding potential of −70 mV. Liquid junction potentials were calculated using pClamp 10.6 and a value of 14 mV was obtained (based on ref. [56]); the applied voltages were corrected offline in every trace. Both of the experiments were recorded in continuous gap-free by 2 min. Only neurons that had a stable resting membrane potential, showed spikes activity and stable access resistance were included in the statistical analysis. The IGOR Pro software (Wave metrics) was used to create all the electrophysiological figures and to perform statistical tests. Analyses were performed on traces that showed frequencies of action potentials higher than 0.2 Hz. For Total inter-burst time calculation, we considered a burst as a train event of at least 4 consecutive action potentials with <4 s ISI. The times among bursts were added and reported as total inter-burst time.

#### Statistical analysis

Results are shown as mean ± SEM. All statistical analyses were conducted using GraphPad Prism v.6 (San Diego, CA) and MATLAB (The Mathworks, Natick, USA). The two-tailed unpaired Student’s *t*-test was used to compare two normally distributed sample groups, and equality of variances was tested through the *F* test, while the Wilcoxon–Mann–Whitney *U* test and Kruskal–Wallis test was used to compare two or more than two non-normally distributed sample groups, respectively. When more than two groups were compared, one-way ANOVA or repeated measures ANOVA followed by Bonferroni’s post-hoc multiple comparison tests were performed to assess significance as indicated in figure legends, and equality of variances tested through the Brown–Forsythe’s and Bartlett’s test. A *p*-value < 0.05 was considered significant. No predictive statistical methods were used to predetermine sample sizes; however, we adopted sample sizes (indicated in figure legends) in the same range as those previously reported in the literature for similar experiments. The ROUT method with *Q* = 1% was used to identify outliers for exclusion from analysis. No randomization method was followed to allocate samples/animals to the various experimental groups. Investigators were not blinded to group allocation but were blinded when assessing the outcome of the experiments.

## Supplementary information

Supplemental Material

Original WB images

checklist

## Data Availability

The datasets used and/or analyzed during the current study are available from the corresponding author on reasonable request.

## References

[1] Bao Z, Wang Y, Wang Q, Fang S, Shan X, Wang J (2021). Intratumor heterogeneity, microenvironment, and mechanisms of drug resistance in glioma recurrence and evolution. Front Med.

[2] Huberfeld G, Vecht CJ (2016). Seizures and gliomas—towards a single therapeutic approach. Nat Rev Neurol.

[3] Samudra N, Zacharias T, Plitt A, Lega B, Pan E (2019). Seizures in glioma patients: an overview of incidence, etiology, and therapies. J Neurol Sci.

[4] Weyer-Jamora C, Brie MS, Luks TL, Smith EM, Braunstein SE, Villanueva-Meyer JE (2021). Cognitive impact of lower-grade gliomas and strategies for rehabilitation. Neurooncol Pract.

[5] Ius T, Pauletto G, Isola M, Gregoraci G, Budai R, Lettieri C (2014). Surgery for insular low-grade glioma: predictors of postoperative seizure outcome. J Neurosurg.

[6] Ius T, Pauletto G, Tomasino B, Maieron M, Budai R, Isola M (2020). Predictors of postoperative seizure outcome in low grade glioma: from volumetric analysis to molecular stratification. Cancers (Basel).

[7] Venkataramani V, Tanev DI, Strahle C, Studier-Fischer A, Fankhauser L, Kessler T (2019). Glutamatergic synaptic input to glioma cells drives brain tumour progression. Nature.

[8] Venkatesh HS, Morishita W, Geraghty AC, Silverbush D, Gillespie SM, Arzt M (2019). Electrical and synaptic integration of glioma into neural circuits. Nature.

[9] Zeng Q, Michael IP, Zhang P, Saghafinia S, Knott G, Jiao W (2019). Synaptic proximity enables NMDAR signalling to promote brain metastasis. Nature.

[10] Singh SK, Hawkins C, Clarke ID, Squire JA, Bayani J, Hide T (2004). Identification of human brain tumour initiating cells. Nature.

[11] Bourkoula E, Mangoni D, Ius T, Pucer A, Isola M, Musiello D (2014). Glioma-associated stem cells: a novel class of tumor-supporting cells able to predict prognosis of human low-grade gliomas. Stem Cells.

[12] Andolfi L, Bourkoula E, Migliorini E, Palma A, Pucer A, Skrap M (2014). Investigation of adhesion and mechanical properties of human glioma cells by single cell force spectroscopy and atomic force microscopy. PLoS ONE.

[13] Manini I, Ruaro ME, Sgarra R, Bartolini A, Caponnetto F, Ius T (2019). Semaphorin-7A on exosomes: a promigratory signal in the glioma microenvironment. Cancers (Basel).

[14] Louis DN, Perry A, Wesseling P, Brat DJ, Cree IA, Figarella-Branger D (2021). The 2021 WHO Classification of Tumors of the Central Nervous System: a summary. Neuro Oncol.

[15] van Niel G, D’Angelo G, Raposo G (2018). Shedding light on the cell biology of extracellular vesicles. Nat Rev Mol Cell Biol.

[16] Tankov S, Walker PR (2021). Glioma-derived extracellular vesicles—far more than local mediators. Front Immunol.

[17] Buckingham SC, Campbell SL, Haas BR, Montana V, Robel S, Ogunrinu T (2011). Glutamate release by primary brain tumors induces epileptic activity. Nat Med.

[18] Yuen TI, Morokoff AP, Bjorksten A, D’Abaco G, Paradiso L, Finch S (2012). Glutamate is associated with a higher risk of seizures in patients with gliomas. Neurology.

[19] Armstrong TS, Grant R, Gilbert MR, Lee JW, Norden AD (2016). Epilepsy in glioma patients: mechanisms, management, and impact of anticonvulsant therapy. Neuro Oncol.

[20] Rudà R, Bello L, Duffau H, Soffietti R (2012). Seizures in low-grade gliomas: natural history, pathogenesis, and outcome after treatments. Neuro Oncol.

[21] Sharma KD, Schaal D, Kore RA, Hamzah RN, Pandanaboina SC, Hayar A (2020). Glioma-derived exosomes drive the differentiation of neural stem cells to astrocytes. PLoS ONE.

[22] Garaschuk O, Hanse E, Konnerth A (1998). Developmental profile and synaptic origin of early network oscillations in the CA1 region of rat neonatal hippocampus. J Physiol.

[23] Baruchi I, Volman V, Raichman N, Shein M, Ben-Jacob E (2008). The emergence and properties of mutual synchronization in in vitro coupled cortical networks. Eur J Neurosci.

[24] Hjorth JJJ, Dawitz J, Kroon T, Pires J, Dassen VJ, Berkhout JA (2016). Detection of silent cells, synchronization and modulatory activity in developing cellular networks. Dev Neurobiol.

[25] Yamamoto H, Kubota S, Chida Y, Morita M, Moriya S, Akima H (2016). Size-dependent regulation of synchronized activity in living neuronal networks. Phys Rev E.

[26] Strogatz S. Sync: the emerging science of spontaneous order. UK: Penguin Press Science; 2004

[27] Kuramoto Y. Self-entrainment of a population of coupled non-linear oscillators. In: Araki H, editor. International symposium on mathematical problems in theoretical physics. Berlin/Heidelberg: Springer-Verlag; 1975. 420–2.

[28] Mirollo RE, Strogatz SH (1990). Synchronization of pulse-coupled biological oscillators. SIAM J Appl Math.

[29] Pecora LM, Carroll TL (1998). Master stability functions for synchronized coupled systems. Phys Rev Lett.

[30] Palacios ER, Isomura T, Parr T, Friston K (2019). The emergence of synchrony in networks of mutually inferring neurons. Sci Rep.

[31] Brunel N (2000). Dynamics of sparsely connected networks of excitatory and inhibitory spiking neurons. J Comput Neurosci.

[32] Teeter C, Iyer R, Menon V, Gouwens N, Feng D, Berg J (2018). Generalized leaky integrate-and-fire models classify multiple neuron types. Nat Commun.

[33] Li X, Spelat R, Bartolini A, Cesselli D, Ius T, Skrap M (2020). Mechanisms of malignancy in glioblastoma cells are linked to mitochondrial Ca^2+^ uniporter upregulation and higher intracellular Ca^2+^ levels. J Cell Sci.

[34] Hessvik NP, Llorente A (2018). Current knowledge on exosome biogenesis and release. Cell Mol Life Sci.

[35] Vishnubhatla I, Corteling R, Stevanato L, Hicks C, Sinden J (2014). The development of stem cell-derived exosomes as a cell-free regenerative medicine. J Circ Biomark.

[36] Théry C, Witwer KW, Aikawa E, Alcaraz MJ, Anderson JD, Andriantsitohaina R (2018). Minimal information for studies of extracellular vesicles 2018 (MISEV2018): a position statement of the International Society for Extracellular Vesicles and update of the MISEV2014 guidelines. J Extracell Vesicles.

[37] Mallawaaratchy DM, Hallal S, Russell B, Ly L, Ebrahimkhani S, Wei H (2017). Comprehensive proteome profiling of glioblastoma-derived extracellular vesicles identifies markers for more aggressive disease. J Neurooncol.

[38] Yamaguchi H, Lorenz M, Kempiak S, Sarmiento C, Coniglio S, Symons M (2005). Molecular mechanisms of invadopodium formation: the role of the N-WASP–Arp2/3 complex pathway and cofilin. J Cell Biol.

[39] Buffolo F, Petrosino V, Albini M, Moschetta M, Carlini F, Floss T (2021). Neuroinflammation induces synaptic scaling through IL-1β-mediated activation of the transcriptional repressor REST/NRSF. Cell Death Dis.

[40] Santello M, Bezzi P, Volterra A (2011). TNFα controls glutamatergic gliotransmission in the hippocampal dentate gyrus. Neuron.

[41] Secomandi N, Franceschi Biagioni A, Kostarelos K, Cellot G, Ballerini L (2020). Thin graphene oxide nanoflakes modulate glutamatergic synapses in the amygdala cultured circuits: exploiting synaptic approaches to anxiety disorders. Nanomedicine.

[42] Molyneaux BJ, Arlotta P, Menezes JRL, Macklis JD (2007). Neuronal subtype specification in the cerebral cortex. Nat Rev Neurosci.

[43] Charlesworth P, Cotterill E, Morton A, Grant SGN, Eglen SJ (2015). Quantitative differences in developmental profiles of spontaneous activity in cortical and hippocampal cultures. Neural Dev.

[44] Sumi T, Yamamoto H, Hirano-Iwata A (2020). Suppression of hypersynchronous network activity in cultured cortical neurons using an ultrasoft silicone scaffold. Soft Matter.

[45] Morin FO, Takamura Y, Tamiya E (2005). Investigating neuronal activity with planar microelectrode arrays: achievements and new perspectives. J Biosci Bioeng.

[46] Sanchez-Vives MV, McCormick DA (2000). Cellular and network mechanisms of rhythmic recurrent activity in neocortex. Nat Neurosci.

[47] Djemil S, Chen X, Zhang Z, Lee J, Rauf M, Pak DTS (2020). Activation of nicotinic acetylcholine receptors induces potentiation and synchronization within in vitro hippocampal networks. J Neurochem.

[48] Plumbly W, Brandon N, Deeb TZ, Hall J, Harwood AJ (2019). L-type voltage-gated calcium channel regulation of in vitro human cortical neuronal networks. Sci Rep.

[49] Chen J, Fei X, Wang J, Cai Z (2020). Tumor-derived extracellular vesicles: regulators of tumor microenvironment and the enlightenment in tumor therapy. Pharm Res.

[50] Rouillard ME, Sutter PA, Durham OR, Willis CM, Crocker SJ (2021). Astrocyte-derived extracellular vesicles (ADEVs): deciphering their influences in aging. Aging Dis.

[51] Nieland L, Morsett LM, Broekman MLD, Breakefield XO, Abels ER (2021). Extracellular vesicle-mediated bilateral communication between glioblastoma and astrocytes. Trends Neurosci.

[52] Xie Y, Bergström T, Jiang Y, Johansson P, Marinescu VD, Lindberg N (2015). The human glioblastoma cell culture resource: validated cell models representing all molecular subtypes. EBioMedicine.

[53] Xiao M, Li X, Song Q, Zhang Q, Lazzarino M, Cheng G (2018). A fully 3D interconnected graphene-carbon nanotube web allows the study of glioma infiltration in bioengineered 3D cortex-like networks. Adv Mater.

[54] Mahmud M, Pulizzi R, Vasilaki E, Giugliano M (2014). QSpike tools: a generic framework for parallel batch preprocessing of extracellular neuronal signals recorded by substrate microelectrode arrays. Front Neuroinform.

[55] Quiroga RQ, Nadasdy Z, Ben-Shaul Y (2004). Unsupervised spike detection and sorting with wavelets and superparamagnetic clustering. Neural Comput.

[56] Barry PH (1994). JPCalc, a software package for calculating liquid junction potential corrections in patch-clamp, intracellular, epithelial and bilayer measurements and for correcting junction potential measurements. J Neurosci Methods.

