# Supplementary material for: The dual action of glioma-derived exosomes on neuronal activity: synchronization and disruption of synchrony
Source: Cell Death Dis. 2022 Aug 13;13(8):705. doi: 10.1038/s41419-022-05144-6 (PMC9376103; doi:10.1038/s41419-022-05144-6)
Supplement: Supplementary file 1 — Supplemental Material [file 41419_2022_5144_MOESM1_ESM.docx]

**The dual action of glioma-derived exosomes on neuronal activity: synchronization and disruption of synchrony**

*Renza Spelat^1^*, Nie Jihua^1^*, Cesar Adolfo Sánchez Triviño^1^*, Simone Pifferi^1^*, Diletta Pozzi^1^, Matteo Manzati^1^, Simone Mortal^1,2^, Irene Schiavo^1^, Federica Spada^1^, Melania Zanchetta^1^, Tamara Ius^3^, Ivana Manini^4^, Irene Giulia Rolle^4^, Pietro Parisse^2^, Ana P. Millán^5^, Ginestra Bianconi^6,7^, Fabrizia Cesca^8^, Michele Giugliano^1^, Anna Menini^1^, Daniela Cesselli^4^, Miran Skrap^9^, Vincent Torre^1,2,10^+*

*^1^International School for Advanced Studies (SISSA), via Bonomea 265, Trieste 34136, Italy*

*^2^Institute of Materials (IOM-CNR), Area Science Park, Basovizza, 34149, Trieste, Italy*

*^3^Neurosurgery Unit, Department of Neurosciences, Santa Maria della Misericordia University Hospital, 33100 Udine, Italy*

*^4^Università degli studi di Udine, Istituto di Anatomia Patologica, ASUIUD, Italy*

*^5^Amsterdam UMC, Vrije Universiteit Amsterdam, Department of Clinical Neurophysiology and MEG Center, Amsterdam Neuroscience, De Boelelaan 1117, Amsterdam, The Netherlands*

*^6^School of Mathematical Sciences, Queen Mary University of London, Mile End Road, E1 4NS, London,UK*

*^7^Alan Turing Institute, The British Library, 96 Euston Road, London UK*

*^8^Department of Life Sciences, University of Trieste, 34127 Trieste, Italy*

*^9^SOC Neurochirurgia. Az. Ospedaliera Sanitaria Integrata. Udine, Italy*

*^10^Biovalley Systems & Solutions S.r.l., 34148 Trieste, Italy*

**equally contributed*

*+corresponding author: email:* [*torre@sissa.it*](mailto:torre@sissa.it)

Running title: glioma-derived exosomes and network synchrony

Keywords: glioma, exosomes, network synchrony, neuronal excitability, calcium imaging

**Supplementary Information**


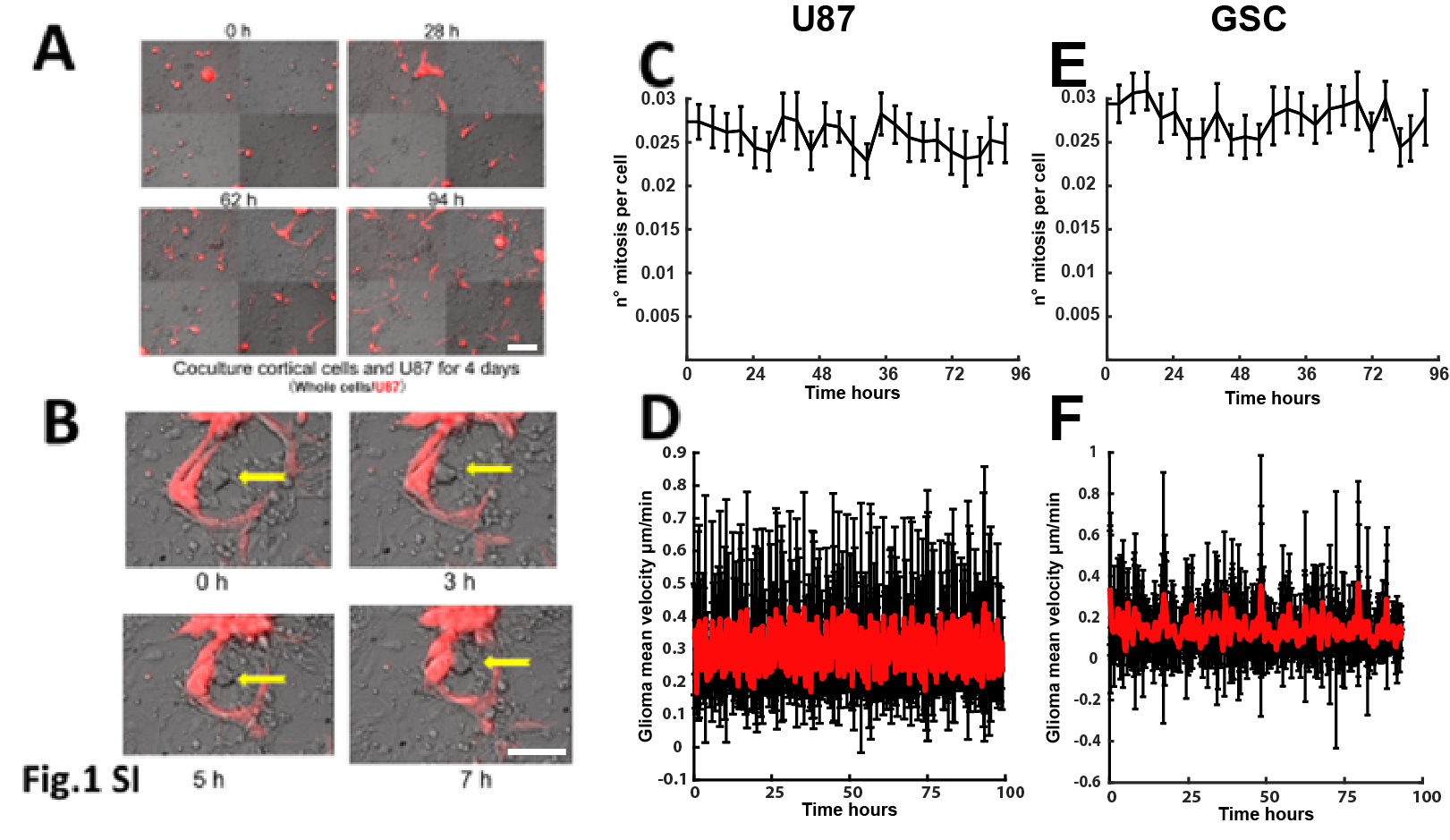


**Figure S1**: *Dynamics of glioma cells co-cultured with primary cortical neurons*. U87 cells (visible because of their red fluorescence) were added to DIV3 cortical neuron cultures and the dynamics of co-cultures followed over time. (**A,B**) Representative images of co-cultures at various time points after U87 addition, as indicated. Yellow arrows in (B) indicate a neuron that is dragged along by a neighboring glioma cell. Scale bars: 20 μm. (**C**, **E**) Rate of replication and (**D**, **F**) mean velocity of U87 cells and GSCs, as indicated (n=3). Graphs show mean ± sem.


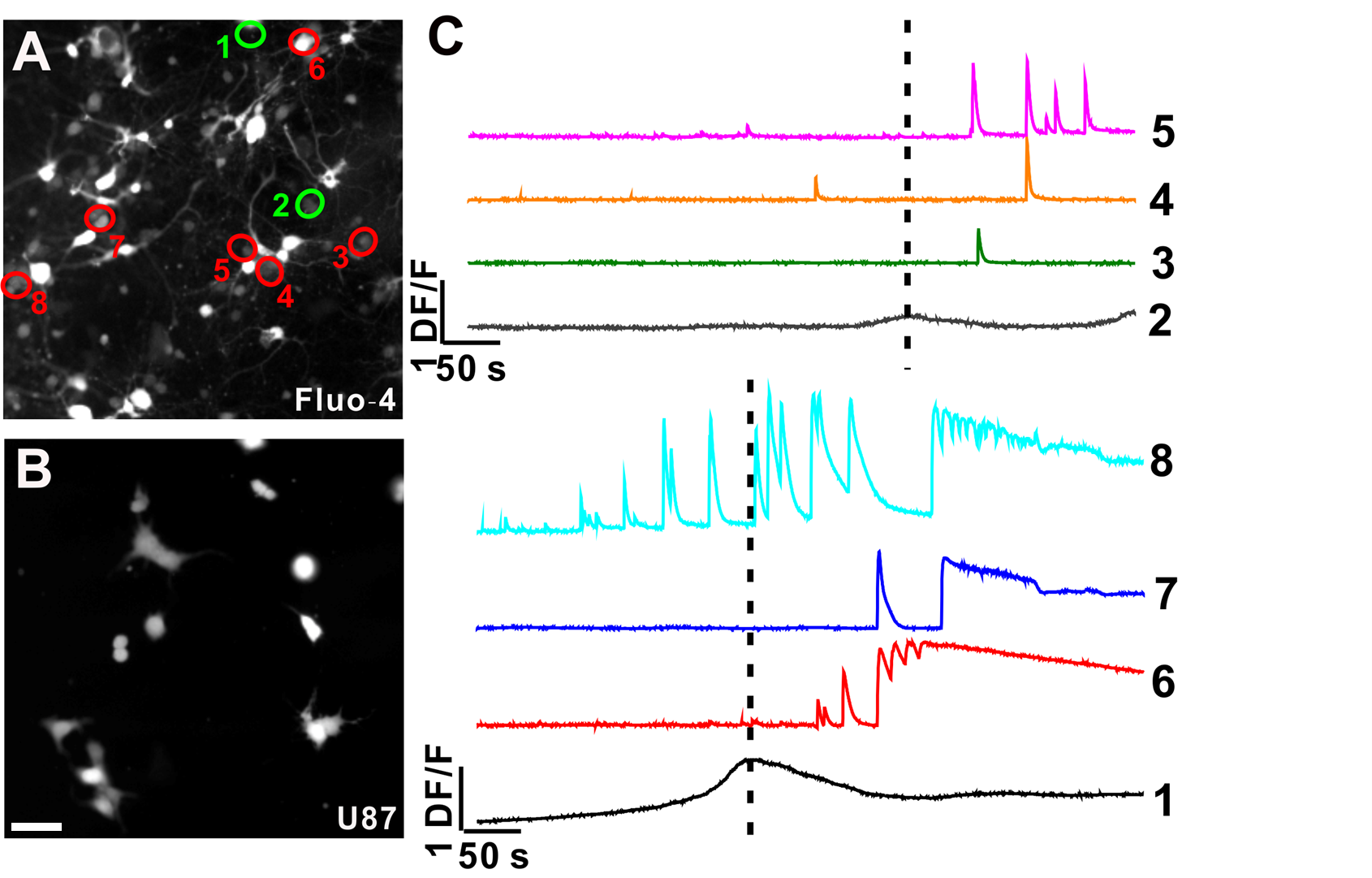


**Figure S2**: *Impact of U87 Ca^2+^ waves on co-cultured neuron activity.* *Left*: representative images of Fluo-4 loaded neurons (top) co-cultured with mCherry-positive U87 glioma cells (bottom). Regions of interest (ROIs) used for detecting the Ca^2+^ transients shown on the right are shown: green circles for glioma cells, red circles for neurons. Scale bar, 20 μm. A Ca^2+^ wave occurring in glioma cells (indicated by the dashed lines) is followed by an increase in firing of neighboring neurons with a delay of 50-100 sec.


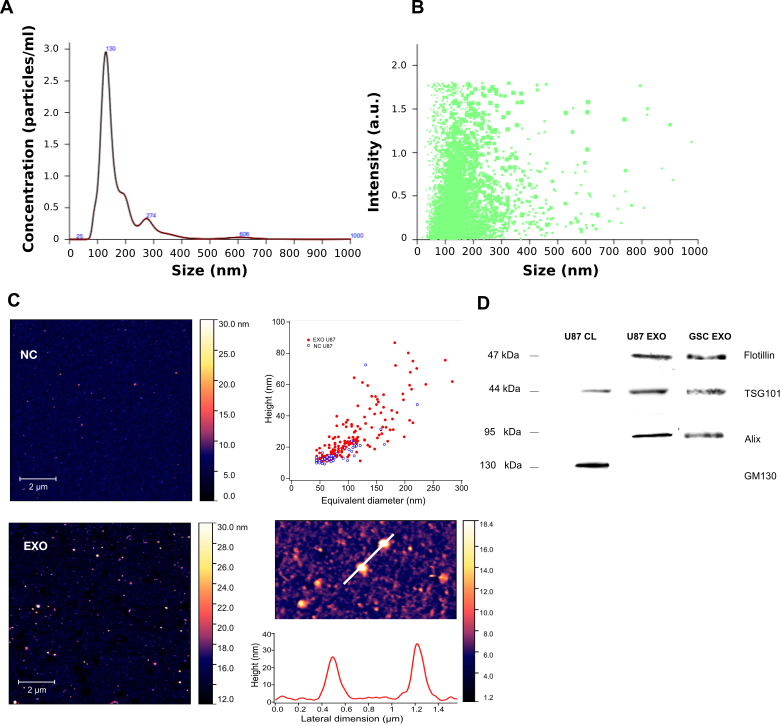


**Figure S3:** *U87 and GSC exosomes characterization.* (**A, B**) The size and concentration of exosomes was analyzed by NTA. The graph in (A) represents the distribution of the exosomes based on their size (nm) and relative concentration (particles/ml), while in (B) the distribution of exosome size (nm) with respect to the relative intensity expressed in arbitrary units (a.u.) is reported. (**C**) *Left*: AFM images show the presence of nano-particles with dimensions ranging from 50 to 150 nm (mean diameter +/- sd = 100 +/-50 nm) in exosome preparations (EXO), as shown in the higher magnification image (4 x 2 μm^2^, *bottom right panel*), with the corresponding line profile. Results obtained from the control sample (NC, negative control) also evidenced the presence of few particles but, as evident in the distribution of height and diameter reported in the scatter plot on the *top right*, with a significantly smaller size distribution. (**D**) Characterization of U87 and GSC exosomes by western blotting. 30 μg of total U87 cell lysate (CL), U87- and GSC-derived exosomes (EXO) were analyzed by SDS-PAGE and western blotting. Antibodies for Flotillin 1, TGS101 and Alix were used to detect exosome markers, while GM130 was present only in U87 cell lysates.


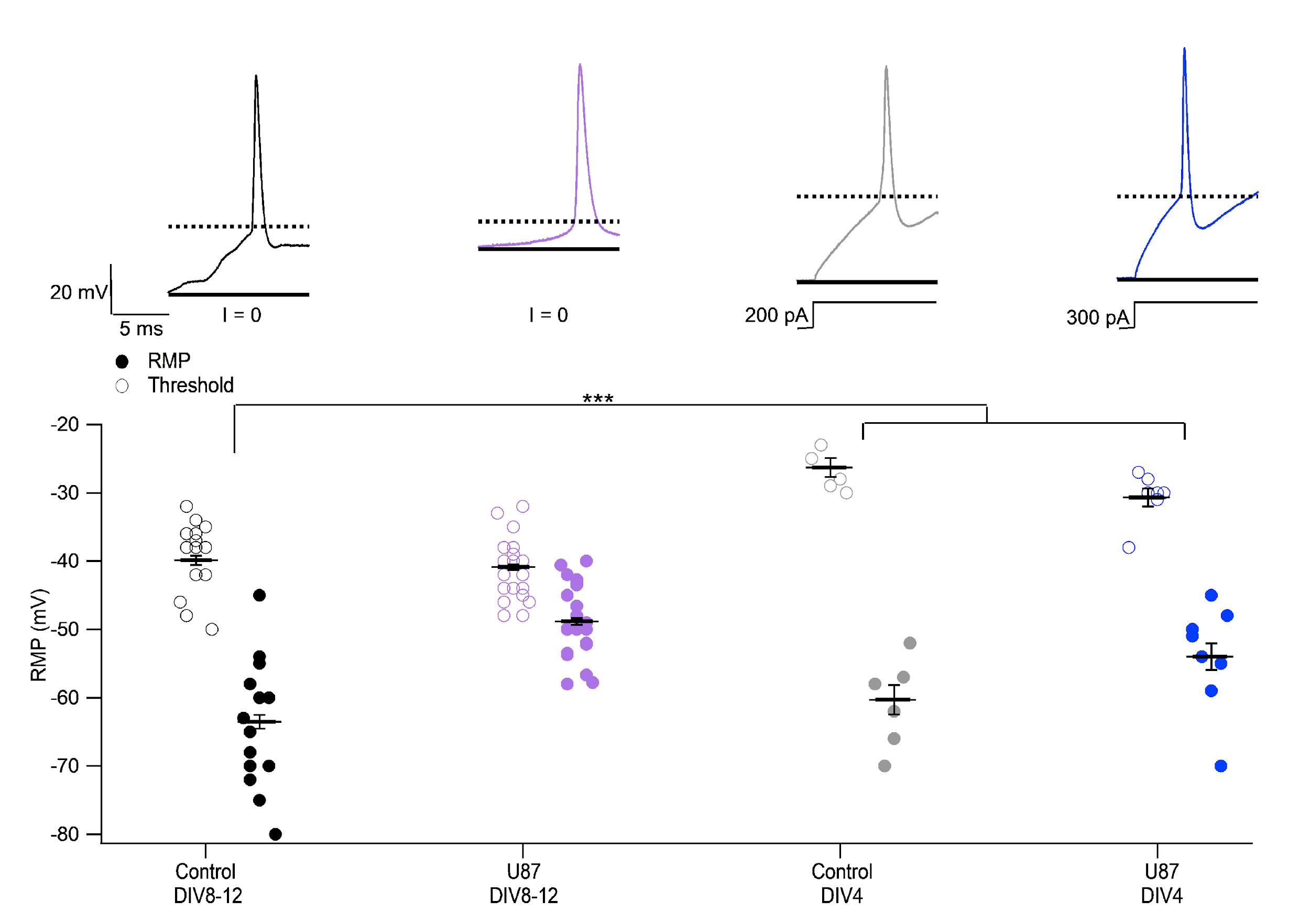


**Figure S4**: *Impact of U87 exosome treatment on RMP and AP threshold in young and mature hippocampal neurons.* *Upper panel*: representative action potential traces from control (black traces) and U87 exosome-treated (purple traces) 8-12 DIV neurons and from control (gray traces) and U87 exosome-treated (blue traces) 4 DIV neurons. Young neurons are immature, and activity is triggered upon injection of 200 to 300 pA current (no spikes were detected during spontaneous activity recordings at 4 DIV, I=0). *Bottom panel*: resting membrane potential (RMP, full circles) and threshold to trigger AP (empty circles) in the same experimental groups. 4 DIV neurons are characterized by a more depolarized RMP (4 DIV: Control -60.83 ± 2.55, U87 treatment -54 ± 2.78 mV; 8-12 DIV: Control -63.53 ± 2.92 mV, U87 treatment -49.083 ± 1.123 mV as in Figure 4C). Immature cells have a very positive AP threshold (Control: -30.67 ± 1.97 mV; U87 treatment: -26.25 ± 1.66 mV) that is statistically different from the mature control cells (-48.83 ± 2.33 mV). Wilcoxon-Mann-Whitney U test, ***p<0.001, n. cells as in Fig. 3, main text.


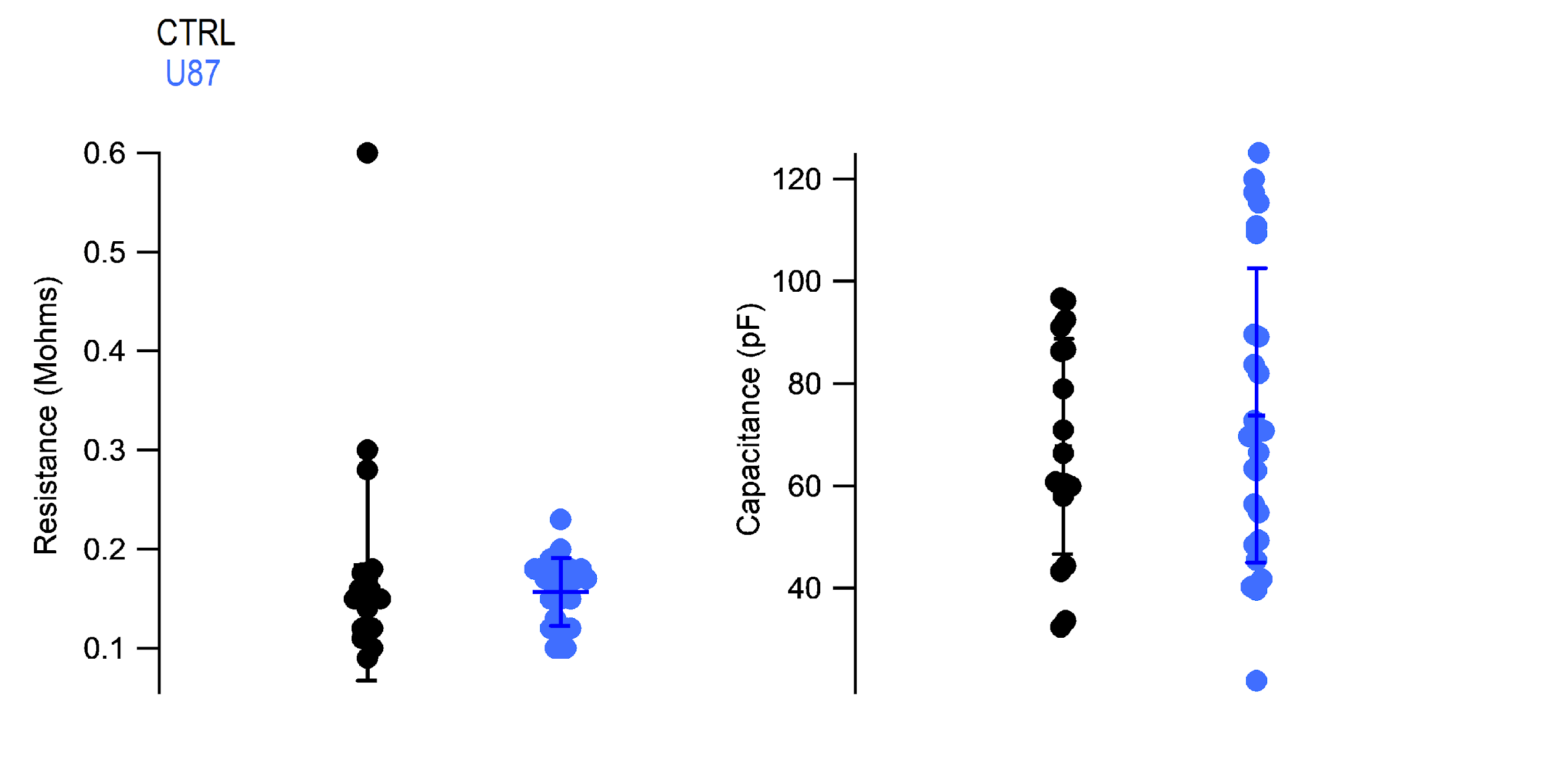


**Figure S5**: *Impact of U87 exosome treatment on membrane resistance and capacitance in young and mature hippocampal neurons.* Measurement of membrane resistance (*left*) and capacitance (*right*) by applying negative step of 5 mV in whole-cell voltage-clamp modality. Control and U87-treated cells do not show statistically significant differences. p>0.05 Wilcoxon-Mann-Whitney U test, n. cells as in Figure 3, main text.

**Figure S6**: *Human astrocytes-derived exosomes do not change the electrophysiological features of hippocampal neurons*. 7-12 DIV neurons treated with exosomes derived from human astrocytes (**A**) show electrophysiological properties (RMP and firing frequency) comparable to control neurons treated with extraction medium (**B**). *Left*: Spike distribution from 6 cells recorded in current clamp configuration, showing a typical low bursting; *Middle*: representative traces after 24 h of treatment with human astrocytes-derived exosomes / extraction medium; *Right*: RMP and Action potential Frequency have a similar distribution to control cells (RMP: HA = -65.4 ± 2.6 mV; Control = -63.5 ± 2 mV; Frequency: HA = 0.15 ± 0.08 Hz; Control = 0.3 ± 0.12 Hz).


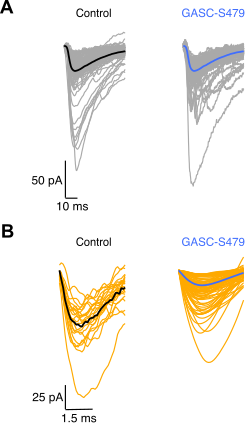


**Figure S7**: *Impact of exosome treatment on GABAergic and glutamatergic events.* Overlay of single GABAergic and glutamatergic events detected for a representative control and exosome-treated cell. (**A**) GABA events (gray), (**B**) glutamatergic events (orange); average event trace is showed in blue for exosome-treated cells and in black for Control cells (*p < 0.05 Kolmogorov-Smirnov test).

**In-silico simulations**

In order to obtain a better insight on the mechanisms of synchronization and disruption of synchrony, we investigated simple mechanisms which could initiate these changes of dynamical behavior. To reproduce the experimentally observed phenomenology we have considered the following leaky integrate and fire (IF) neuronal dynamics.

*The network model*

We modelled the neuronal network as a random geometric network formed by a set of $N$ neurons placed randomly on a 2d square of side length $l=1,$with each node connected to all the nodes at distance $d<d_{c}$, where $d_{c}$ determines the connectivity of the network. An example of the resulting network is shown in figure S8.


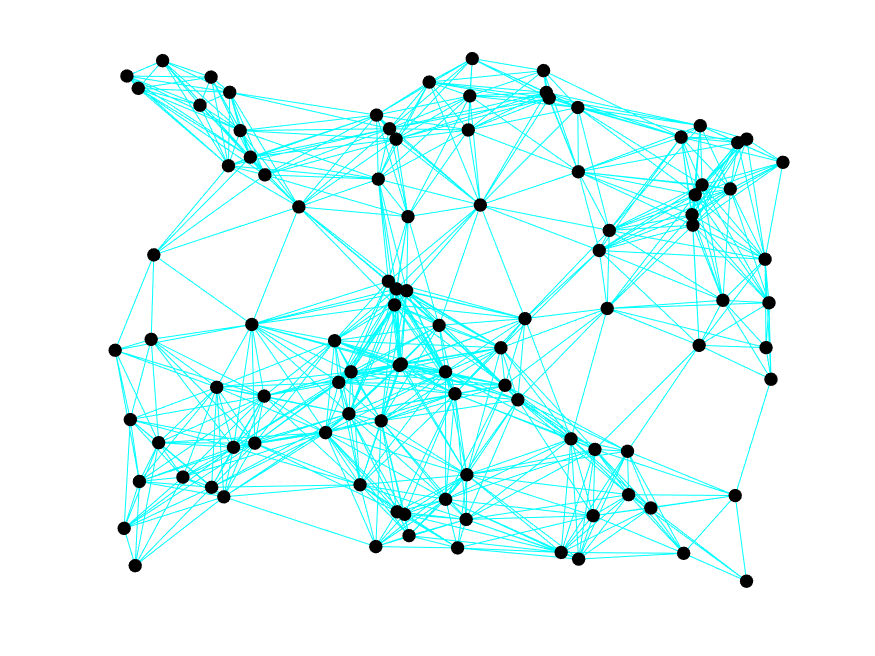


**Figure S8:** Exemplary network with $d_{c}=0.25$.

*The integrate-and-fire (IF) model*

The neuronal dynamics follows the leaky IF model:

$$\frac{dV_{i}}{dt}=-\frac{V_{i}}{\tau_{i}}+{R(I}_{i}^{syn}+I_{0}+\varepsilon\eta_{i}\left( t \right))$$

where $V_{i}$ is the voltage, R=1G\Ohm is the membrane characteristic resistance constant and $\tau_{i}$ is the membrane time constant of neuron $i$*,* $I_{i}^{syn}$ indicates the incoming synaptic current arriving at neuron $i$, I_0_ indicates the external current,$\varepsilon$ the strength of the random noise and $\eta_{i}(t)$ is a Gaussian noise with average zero and standard deviation one, i.e.$\eta_{i}\sim N(0,1)$.

The incoming synaptic current $I_{i}^{syn}$ arriving at neuron $i$ from all its neighbors is given by

$I_{i}^{syn}=\sigma\sum_{j=1}^{N} w_{ij}\sum_{n} \delta(t-t_{j}^{(n)})$.

where $t_{j}^{(n)}$ indicates the time of the $n$-th spike of neuron $j,$ and $\delta(x)$ is the delta function.

Here $\sigma$ indicates the strength of the synaptic coupling and $w_{ij}$ indicates the generic element of the weighted and signed adjacency matrix of the network which is positive $w_{ij}=w_{+}>0$if $j$ is an excitatory neuron of $i$, is negative $w_{ij}=w_{-}<0$ if $j$ is an inhibitory neuron of $i$ and zero if there is no connection between neurons $i$ and $j$.

Once $V_{i}$reaches the firing threshold$V_{th}$, the neuron fires a spike and its voltage is reset to 0 for a time $T_{ref}$ (with $T_{ref}>0$) to account for refractory effects after firing. In order to prevent non-realistic arbitrarily large hyperpolarization, a minimum voltage value is set to $V_{min}=-3V_{th}$.

The initial condition of the integrate-and-fire dynamics is a condition where each neuron fires with probability $p_{activation}$.

Note that in order to satisfy detailed balance the ratio ${|w}_{-}|/|w_{+}|$ should be equal to the ratio between excitatory neurons and inhibitory neurons. Specifically, in all our simulation we consider neuronal networks formed by 80% of excitatory neurons and 20% of inhibitory ones with $w_{-}=-4,w_{+}=1$ so that there are 4 times more excitatory neurons, but inhibitory synapses are 4 times stronger.

Moreover, we have taken $\varepsilon=\sqrt{\sigma} ,$ $T_{ref}=2ms$, activation probability $p_{activation}=0.5$ and we have drawn the intrinsic times $\tau_{i}$ randomly from a uniform distribution in the interval *[5,6] ms.* All simulations are carried out in Matlab2021 using a Euler-Maruyama algorithm with integration step *dt=0.1*. Before recording the neuronal dynamics, we allowed the evolution of the system for a transient time corresponding to 400 ms.
