# Supplementary material for: The dual action of glioma-derived exosomes on neuronal activity: synchronization and disruption of synchrony
Source: Cell Death Dis. 2022 Aug 13;13(8):705. doi: 10.1038/s41419-022-05144-6 (PMC9376103; doi:10.1038/s41419-022-05144-6)
Supplement: Supplementary file 2 — Original WB images [file 41419_2022_5144_MOESM2_ESM.docx]

**The dual action of glioma-derived exosomes on neuronal activity: synchronization and disruption of synchrony**

*Renza Spelat^1^*, Nie Jihua^1^*, Cesar Adolfo Sánchez Triviño^1^*, Simone Pifferi^1^*, Diletta Pozzi^1^, Matteo Manzati^1^, Simone Mortal^1,2^, Irene Schiavo^1^, Federica Spada^1^, Melania Zanchetta^1^, Tamara Ius^3^, Ivana Manini^4^, Irene Giulia Rolle^4^, Pietro Parisse^2^, Ana P. Millán^5^, Ginestra Bianconi^6,7^, Fabrizia Cesca^8^, Michele Giugliano^1^, Anna Menini^1^, Daniela Cesselli^4^, Miran Skrap^9^, Vincent Torre^1,2,10^+*

*^1^International School for Advanced Studies (SISSA), via Bonomea 265, Trieste 34136, Italy*

*^2^Institute of Materials (IOM-CNR), Area Science Park, Basovizza, 34149, Trieste, Italy*

*^3^Neurosurgery Unit, Department of Neurosciences, Santa Maria della Misericordia University Hospital, 33100 Udine, Italy*

*^4^Università degli studi di Udine, Istituto di Anatomia Patologica, ASUIUD, Italy*

*^5^Amsterdam UMC, Vrije Universiteit Amsterdam, Department of Clinical Neurophysiology and MEG Center, Amsterdam Neuroscience, De Boelelaan 1117, Amsterdam, The Netherlands*

*^6^School of Mathematical Sciences, Queen Mary University of London, Mile End Road, E1 4NS, London,UK*

*^7^Alan Turing Institute, The British Library, 96 Euston Road, London UK*

*^8^Department of Life Sciences, University of Trieste, 34127 Trieste, Italy*

*^9^SOC Neurochirurgia. Az. Ospedaliera Sanitaria Integrata. Udine, Italy*

*^10^Biovalley Systems & Solutions S.r.l., 34148 Trieste, Italy*

**equally contributed*

*+corresponding author: email:* [*torre@sissa.it*](mailto:torre@sissa.it)

Running title: glioma-derived exosomes and network synchrony

Keywords: glioma, exosomes, network synchrony, neuronal excitability, calcium imaging

**Original western blots**

**Figure 4 B/D**


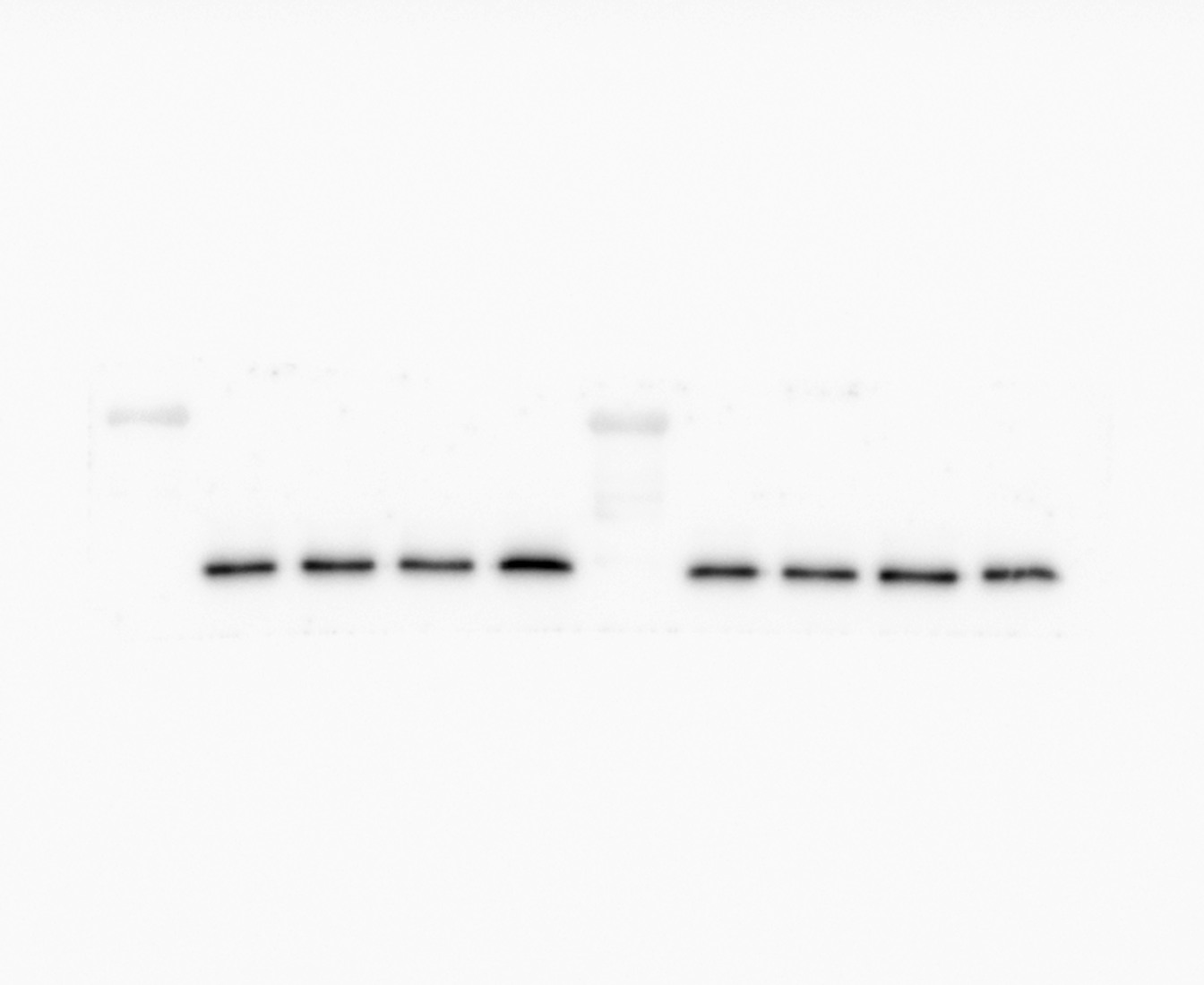


ARP3

47 kDa -

Ctr 7-12DIV Exo 7-12DIV Ctr 4DIV Exo 4DIV


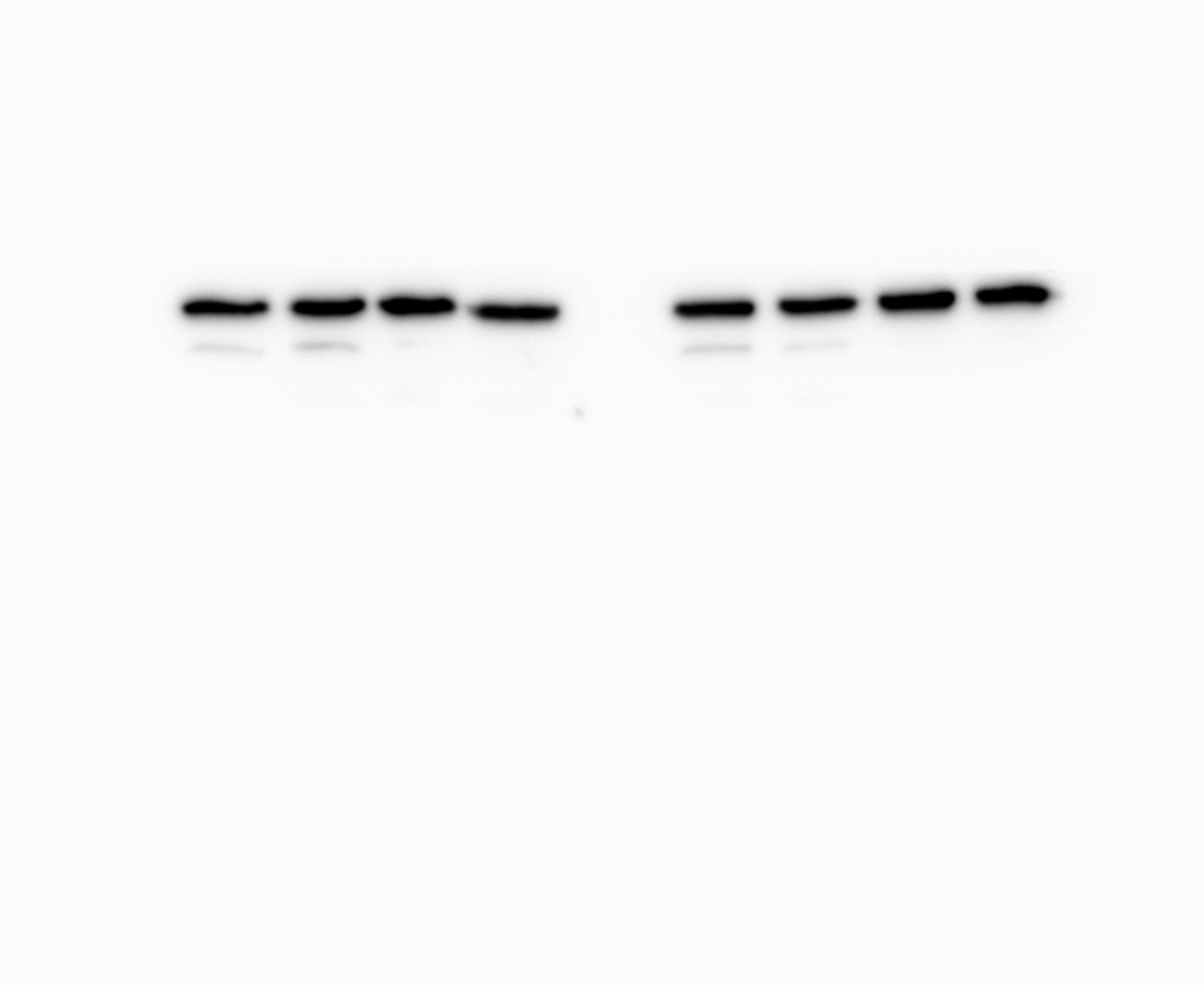


Gapdh

37 kDa -

Ctr 7-12DIV Exo 7-12DIV Ctr 4DIV Exo 4DIV

**Figure S3:**

*for TSG101 and Flotillin, the original membrane was cut, we therefore provide only the original acquisition of the immunoreactivity.

WB on U87 exosomes


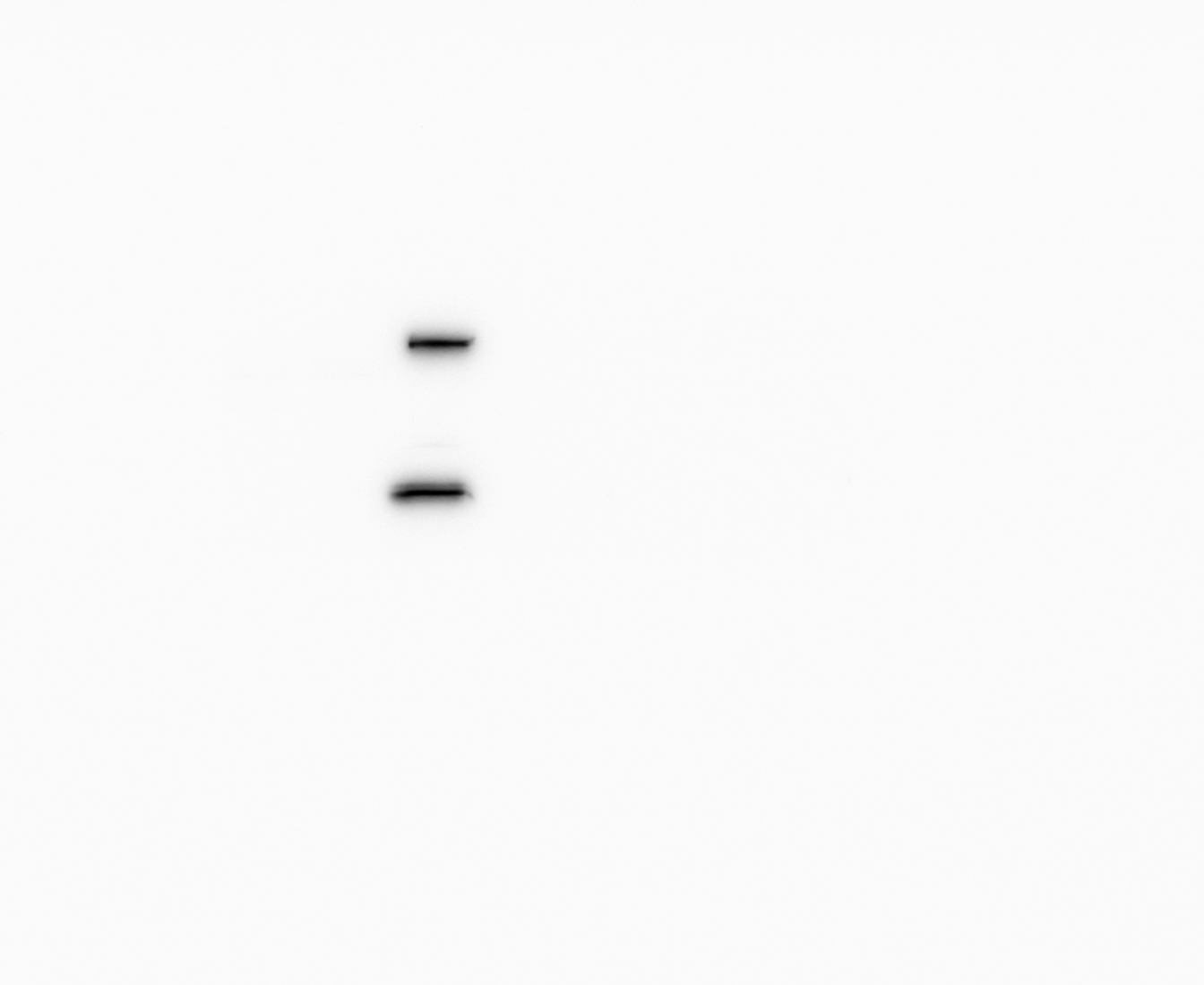


130 kDa -

GM130
 U87 CL U87 Exo


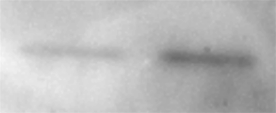


44 kDa -

TSG101

U87 CL U87 Exo


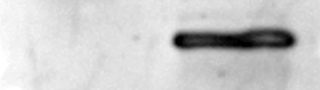


47 kDa -

Flotillin

U87 CL U87 Exo


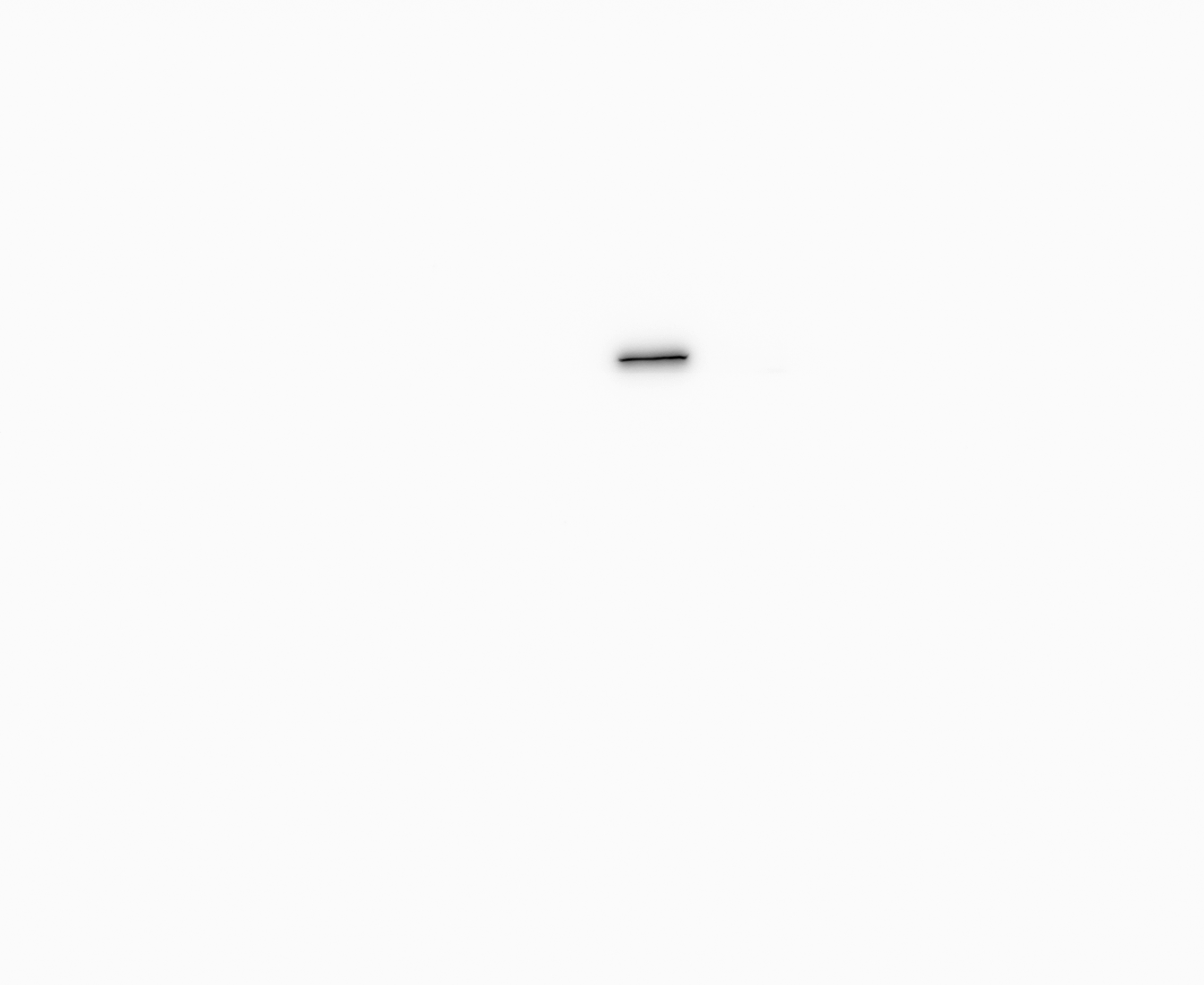


95 kDa -

Alix

U87 CL U87 Exo

WB on GSC exosomes


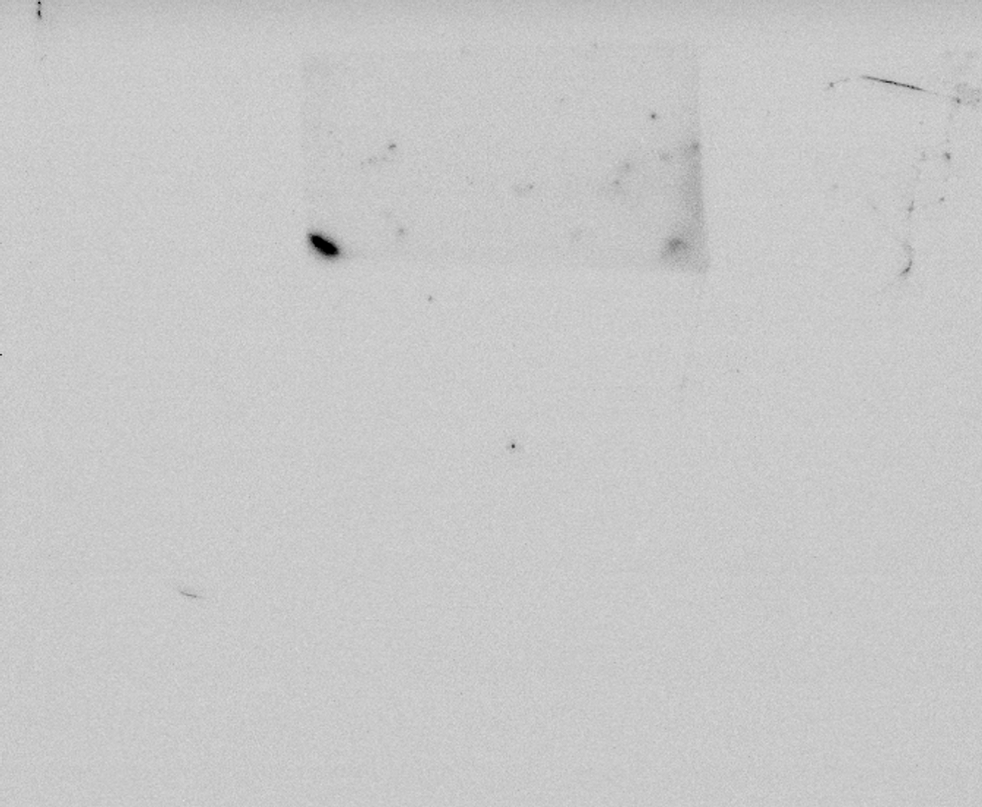


130kDa -

GM130

MW GSC EXO


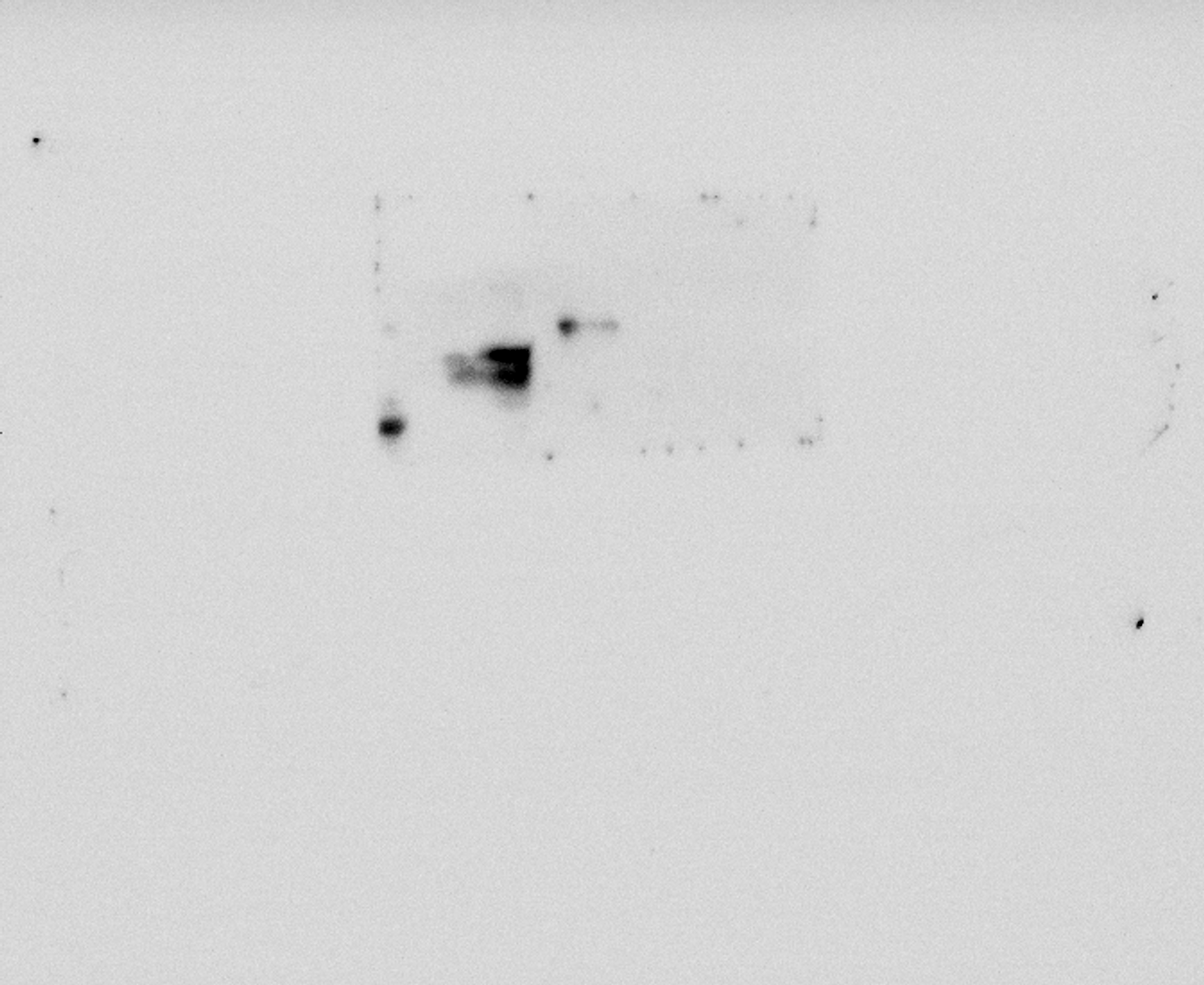


TSG101

44 kDa -

35 kDa -

MW GSC EXO


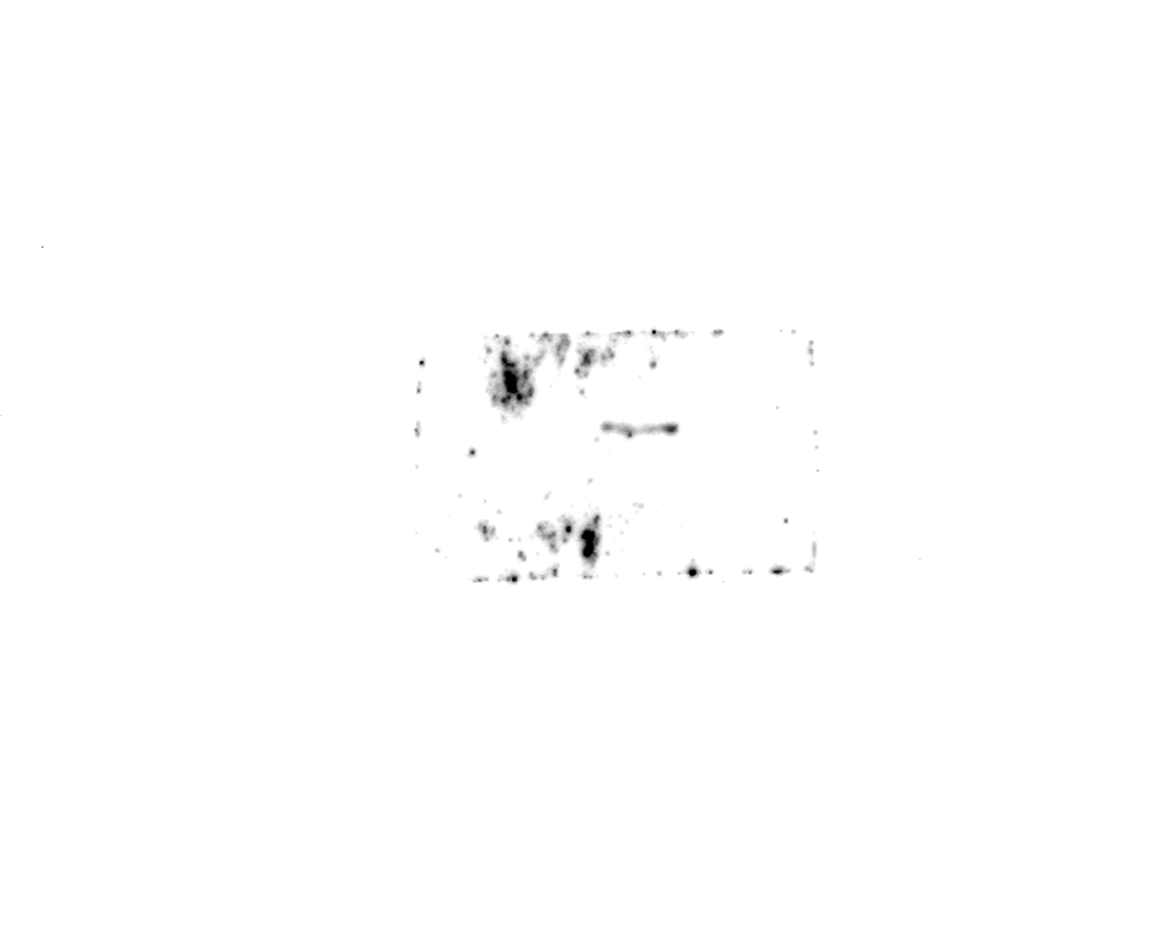


47 kDa -

Flotillin

MW GSC EXO





Alix

95 kDa -

75 kDa -

MW GSC EXO MW
